# Downregulation of m^6^A Reader YTHDC2 Promotes the Proliferation and Migration of Malignant Lung Cells via CYLD/NF-κB Pathway

**DOI:** 10.7150/ijbs.58514

**Published:** 2021-06-22

**Authors:** Jin Wang, Lirong Tan, Beibei Jia, Xiaofan Yu, Ruixin Yao, Nan OUYang, Xueting Yu, Xiyuan Cao, Jian Tong, Tao Chen, Rui Chen, Jianxiang Li

**Affiliations:** 1 Department of Toxicology, School of Public Health, Medicine College, Soochow University, Suzhou, Jiangsu, 215123, China.; 2 Department of Respiratory Medicine, The Second Affiliated Hospital of Soochow University, Suzhou Jiangsu, 215004, China.

**Keywords:** lung cancer, YTHDC2, m^6^A RNA methylation, CYLD, NF-κB pathway

## Abstract

Lung cancer is one of the most common types of carcinoma worldwide. Cigarette smoking is considered the leading cause of lung cancer. Aberrant expression of several YT521-B homology (YTH) family proteins has been reported to be closely associated with multiple cancer types. The present study aims to evaluate the function and regulatory mechanisms of the N6-methyladenosine (m^6^A) reader protein YTH domain containing 2 (YTHDC2) by *in vitro*, *in vivo* and bioinformatics analyses. The results revealed that YTHDC2 was reduced in lung cancer and cigarette smoke-exposed cells. Notably, bioinformatics and tissue arrays analysis demonstrated that decreased YTHDC2 was highly associated with smoking history, pathological stage, invasion depth, lymph node metastasis and poor outcomes. The *in vivo* and* in vitro* studies revealed that YTHDC2 overexpression inhibited the proliferation and migration of lung cancer cells as well as tumor growth in nude mice. Furthermore, YTHDC2 decreased expression was modulated by copy number deletion in lung cancer. Importantly, the cylindromatosis (CYLD)/NF-κB pathways were confirmed as the downstream signaling of YTHDC2, and this axis was mediated by m^6^A modification. The present results indicated that smoking-related downregulation of YTHDC2 was associated with enhanced proliferation and migration in lung cancer cells, and appeared to be regulated by DNA copy number variation. Importantly, YTHDC2 functions as a tumor suppressor through the CYLD/NF-κB signaling pathway, which is mediated by m^6^A modification.

## Introduction

Lung cancer is one of the most common types of carcinoma in the world 1. The two main lung cancer types are small cell lung cancer (SCLC) and non-small cell lung cancer (NSCLC). NSCLC accounts for 85-90% of all lung cancer cases, and its two most common subtypes are lung squamous cell carcinoma (LUSC) and lung adenocarcinoma (LUAD) 2. Despite significant advances in early diagnosis and treatment, the 5-year relative overall survival (OS) of lung cancer patient is <20% 3. Smoking is the main cause of lung cancer of all major histological types, and the risk of lung cancer in persistent smokers is 20-50-fold higher than that of non-smokers 4. A previous study reported that a decreased relative risk and favorable prognosis were found in reformed smokers 5. Numerous candidate biomarkers contribute to the initialization and progress of lung cancer have been reported in bioinformatics analyses 6-8. Therefore, the association between smoking and lung cancer, including the significance of differentially expressed genes caused by smoking, and its role in the development of lung cancer, should be further investigated 9. A previous study suggested that the potency of cigarettes as carcinogens may be due to their ability to induce aberrant DNA methylation and altered gene expression, resulting in cells escaping from apoptosis and undergoing subsequent neoplastic transformation 10, 11.

Previous reports suggested that the dysregulation of N6-methyladenosine (m^6^A) modifications exerts oncogenic and tumor-suppressive effects in the genesis and development of several cancer types, including lung cancer, liver cancer and acute myeloid leukemia 12-14. Several proteins in the YT521-B homology (YTH) family were shown to be capable of selectively binding to and processing precursor RNA containing m^6^A in the nucleus, including YTH N6-Methyladenosine RNA binding protein (YTHDF)1, YTHDF2, YTHDF3, YTH domain containing (YTHDC)1 and YTHDC2, which are considered to be m^6^A reader proteins 15, 16. These readers can promote efficient translation and degradation of specific m^6^A-containing mRNA, causing rapid changes in gene expression profiles 17, 18. The increase in m^6^A methyl-transferase activity was consistently associated with the malignant transformation of cells 19. Among these m^6^A readers, YTHDF1 was reported to be associated with hepatocellular carcinoma (HCC) by promoting the Wnt/β-catenin signaling pathway 20. Several reports demonstrated that YTHDF2 was upregulated in HCC 21 and pancreatic cancer 22. YTHDC2, which is different from the other family members, contains other putative RNA-binding domains in addition to its YTH domain. In addition, YTHDC2 can enhance translation efficiency and decrease the target mRNA abundance by preferentially binding to m^6^A within the consensus motif 23, 24. YTHDC2 plays a vital role in spermatogenesis by interacting with meiosis-specific coiled-coil domain-containing protein, thereby affecting the stability of target transcripts in pre-meiosis 25. However, the precise association between YTHDC2 and cancer remains unclear. It has been reported that upregulated YTHDC2 contributes to the metastasis of colon cancer cells *in vivo* by promoting the translation of cell migration-related genes 26. It has been demonstrated that the downregulation of YTHDC2 can inhibit HCC cell proliferation by reducing the translation of mRNAs involved in cell proliferation 27. However, the role of YTHDC2 in other types of tumors, particularly lung cancer, remains unresolved.

The purpose of this study was to evaluate the expression profile of YTHDC2, a binding-protein of RNA m^6^A methylation, and to determine its *in vitro* effects on cigarette smoke (CS)-induced malignant transformed BEAS-2B cells and lung cancer cell behavior, as well as the clinical relevance and regulatory mechanism of YTHDC2.

## Materials and methods

### Data analysis from The Cancer Genome Atlas (TCGA) and Gene Expression Omnibus (GEO) datasets

The log_2_-transformed RNA-sequencing expression value and copy number alterations of lung cancer in TCGA database, including lung adenocarcinoma (TCGA LUAD) and lung squamous cell carcinoma (TCGA LUSC), were obtained from the University of California Santa Cruz Xena Browser (http://xena.ucsc.edu). Expression of *YTHDC2* mRNA was analyzed based on an online tool, Gene Expression Profiling Interactive Analysis (GEPIA; http://gepia.cancer-pku.cn/index.html), which is a newly developed interactive web server for analyzing the RNA-sequencing expression data of 23 types of cancer and normal samples from TCGA and Genotype-Tissue Expression (GTEx) projects according to the standard processing pipeline 28. Two datasets, GSE32665 29 and GSE19188 30, were download from the GEO database (http://www.ncbi.nlm.nih.gov/geo/) for differential expression analysis. Another two datasets, GSE41271 31 and GSE30219 32, were downloaded for clinicopathological correlation and survival analyses. The characteristics of the datasets used in the present study are displayed in Table S1. At the protein level, the expression of YTHDC2 in normal and malignant lung tissue was reviewed by data mining in the Human Protein Atlas (HPA; http://www.proteinatlas.org), which included 3 normal lung samples and 10 lung cancer samples. Images of YTHDC2 protein immunohistochemistry (IHC) staining in normal lung tissues and lung cancer tissues were downloaded from this database.

### IHC of tissue array

Human lung cancer tissue arrays (cat. no. LAC-1402 and LAC-1403) were purchased from Wuhan Servicebio Technology Co., Ltd. The tissue arrays consisted of 70 pairs of lung cancer and adjacent normal tissues. IHC assay was used to analyze the expression of YTHDC2 in lung cancer and paired normal tissues with an anti-YTHDC2 antibody (1:500 dilusion; ProteinTech Group, Inc.). ImageJ and ImageJ plug-in IHC Profiler were applied for the quantification of IHC staining 33. The positive staining ratio was expressed as the sum of the high, low and positive rates.

### Survival analysis

The association between the mRNA expression levels of the *YTHDC2* gene and the OS of patients with lung cancer was analysed using the online Kaplan-Meier plotter (http://kmplot.com/analysis/) 34. In addition to smoking history, other parameters were set to default values. Survival information and *YTHDC2* expression data of smokers and non-smokers were obtained according to their smoking history. For the survival analysis of the GSE41271, GSE30219 and TCGA lung cancer datasets, the patients were classified into high (≥75%) and low (<75%) *YTHDC2* expression groups according to the % of *YTHDC2* mRNA expression levels. Univariate Cox models were used to calculate the hazard ratios (HRs) and 95% confidence intervals (CIs). Cox analysis was performed using the 'survival' (https://cran.r-project.org/web/packages/survival/index.html) package, and the 'survminer' (https://github.com/kassambara/survminer) package was used to generate Kaplan-Meier survival curves.

### Correlation and enrichment analysis

LinkedOmics (http://linkedomics.org/) is a newly developed web portal based on multi-omics datasets from 32 cancer types in TCGA database 35. The present study applied this portal to identify the genes listed as positively or negatively correlated with *YTHDC2* in the LUAD and LUSC TCGA datasets. These correlated genes were further subjected to enrichment analysis, which was performed using the online enrichment tool DAVID (https://david.ncifcrf.gov) 36. TSGene (https://bioinfo.uth.edu/TSGene/) is a web database for tumour suppressor genes (TSGs) collected from the published literature 37. Human Cancer Metastasis Database (HCMDB; http://hcmdb.i-sanger.com/index) is an integrated database that has annotated multiple potentially metastasis-related genes (MRGs) 38. These two databases were used to identify TSGs and MRGs among the *YTHDC2*-related genes obtained from the LinkedOmics database.

### *In vitro* cell model of CS-induced malignant transformation

Normal human bronchial epithelial cells (BEAS-2B) were obtained from the American Type Culture Collection and used to establish an* in vitro* model of CS-induced malignant transformation, as previously described 10. Briefly, aliquots of exponentially growing 1×10^5^ BEAS-2B cells were plated onto a Transwell membrane (0.4-μm pore; Corning, Inc.). An automatic smoking machine was used to produce CS, which was then pumped into an inhalation chamber where the BEAS-2B cells were directly exposed to CS for 10 min every other day at a smoke concentration of 20% (MED8170A, Tianjin Hope Industry & Trade Co., Ltd). This procedure was performed to expose the cells to CS for 10, 20 and 30 passages, and such cells were referred as experimental S10, S20 and S30 cells, respectively. The unexposed BEAS-2B cells were used as control cells.

### Cell culture and transfection

Human NSCLC H1299 cells were obtained from the American Type Culture Collection. The cells were cultured in Dulbecco's modified Eagle's medium (DMEM) supplemented with 10% fetal bovine serum (FBS) in a humidified incubator with 5% CO₂ at 37 °C. The full-length of the *YTHDC2* coding sequence was amplified using 2XEasyPfu PCR SuperMix (Beijing Transgen Biotech Co., Ltd.) and cloned into a pCDH vector, and the recombinant plasmid was named pCDH-YTHDC2. The design of shRNAs of m^6^A methylases METTL3/14 and demethylases FTO/ALKBH5 relied on the algorithm of an online program BLOCK-iT RNAi Designer (https://rnaidesigner.thermofisher.com/rnaiexpress/). The shRNA oligo pairs were annealed and then inserted into GreenPuro vectors separately. *YTHDC2* small interfering RNA (siRNA) was obtained from Guangzhou RiboBio Co., Ltd. The lentiviruses were packaged according to the manufacturer's protocol. Briefly, supernatants containing YTHDC2 lentivirus generated in 293T cells were collected at 48 and 72 h post-transfection. BEAS-2B, H1299 and S30 cells were transfected with pCDH-YTHDC2 and siRNA using Lipofectamine 6000 (Beyotime Institute of Biotechnology) according to the manufacturer's instructions.

### Tandem mass tagging (TMT) proteomics profiling analysis

Protein samples were extracted from normal BEAS-2B and YTHDC2-knockdown cells. The specific proteomic pipeline is shown in the Supplementary methods. The false discovery rate was adjusted to <1% and the minimum score for modified peptides was set to >40. A ratio >1.2 was considered upregulation, and <0.83 was considered downregulation. The differentially expressed proteins were depicted in the STRING database to generate protein-protein interaction network and identify clusters by plug-in Molecular Complex Detection (MCODE) in Cytoscape (https://cytoscape.org/) 39.

### Reverse transcription-quantitative PCR (RT-qPCR)

Total RNA of samples was isolated using TRIzol^®^ reagent (Invitrogen; Thermo Fisher Scientific, Inc.) according to the manufacturer's protocol. Total RNA (~1.5 μg) from each sample was reverse transcribed into cDNA using a Revert Aid First Strand Complementary DNA Synthesis kit (Thermo Fisher Scientific, Inc.) according to the manufacturer's instructions. RT-qPCR was performed using an RT-qPCR kit (Novoprotein Scientific, Inc.) on a QuantStudio™ 6 Flex Real-Time PCR system (Applied Biosystems; Thermo Fisher Scientific, Inc.). GAPDH was used as a reference. The primer pairs used for qPCR in the study is listed in Table S2. The relative expression of each target was analyzed using the 2^‑ΔΔCt^ method 40.

### Western blot analysis

Total protein was extracted with RIPA buffer (Beyotime, Institute of Biotechnology) and quantified with BCA assay (Beyotime, Institute of Biotechnology), and 20 µg protein was separated on SDS-PAGE and transferred onto a PVDF membrane (MilliporeSigma). Nuclear and cytoplasm proteins were isolated using the Nuclear and Cytoplasmic Protein Extraction kit (CWBio) according to the manufacturer's protocol. After blocking with 5% bovine serum albumin (BSA), the membrane was incubated at 4 °C overnight with specific antibodies against YTHDC2 (1:5,000 dilution, Abcam), E-cadherin (CDH1; 1:1,000 dilution; ProteinTech Group, Inc.), N-cadherin (CDH2; 1:1,000 dilution; ProteinTech Group, Inc.), cylindromatosis (CYLD; 1:1,000 dilution; ProteinTech Group, Inc.), NF-κB P65 (1:1,000 dilution; ProteinTech Group, Inc.) and GAPDH (1:1,000 dilution; Cell Signaling Technology, Inc.). HRP-labeled secondary antibodies were used according to the host species of the primary antibodies. The western blot bands were developed using an ECL substrate and visualized using the GeneTools GBOX system (Syngene International). The intensity of each band was quantified using ImageJ software (Version 1.8.0).

### Cell proliferation assays

For cell cycle analysis, the transfected cells were trypsinized and then washed twice with pre-cold PBS. Cells were fixed in 70% ethanol at -20 °C overnight, washed twice with PBS, and treated with 10 μg/ml RNase A (Beyotime Institute of Biotechnology) and 50 μg/ml propidium iodide (Beyotime Institute of Biotechnology) for 30 min at 37 °C. Cell cycle analysis was performed using a BD FACSCanto™ Cell Analyzer (BD Biosciences), and the percentage of cells in the different phases of the cell cycle was calculated by FlowJo v10.3 software (FlowJo LLC).

For the 5-ethynyl-2'-deoxyuridine (EdU) assay, logarithmic growth stage cells (2×10⁵) were seeded into a 6-well plate with 10 μM EdU reagent for 2 h. After fixing with 4% paraformaldehyde for 30 min, cells were permeabilized with 0.3% Triton X-100 in PBS, and incubated with Click‑iT reaction solution (cat. no. C0075S; Beyotime Institute of Biotechnology). Images were collected at 24 h under an inverted fluorescent microscope and quantitatively analyzed with NIH ImageJ software (Version 1.8.0).

### Immunofluorescence assay

Cells were seeded onto glass coverslips placed in 6-well plates. After fixing with 4% paraformaldehyde for 30 min, the cells were permeabilized with 0.3% Triton X-100 in PBS and blocked with 1% BSA. Next, the cells were incubated with anti-Ki67 polyclonal antibody (1:500 dilution; ProteinTech Group, Inc.) for 1 h at 37 °C. Subsequently, the cells were incubated with an Alexa Fluor^®^ 448-conjugated secondary antibody (1:1,000 dilution; Cell Signaling Technology, Inc.) for 30 min and stained with DAPI (Beyotime Institute of Biotechnology) for 5 min protect from light at room temperature. Images were collected under an inverted fluorescent microscope and quantitatively analyzed with NIH ImageJ software (Version 4.0.4).

### Cell migration assays

For scratch wound healing assay, cells (2×10^5^) were seeded into 6-well plates, cultured at 37 °C and allowed to grow to 100% confluence. Next, a scratch was made in the plate using a P10 pipette tip. The cells were cultured in fresh serum-free DMEM. Images were collected at 0 and 24 h under an inverted microscope (Olympus Corporation) and quantitatively analyzed with NIH ImageJ software (Version 4.0.4).

For the Transwell migration assay, cells (2×10^5^) were seeded in the upper chambers (pore size, 8 μm) of a 6-well plate (Corning, Inc.) with 1 ml serum-free medium. A total of 2 ml complete medium with 10% FBS was added to the lower chambers, and the plate was incubated for 24 h. Next, the cells in the upper surface of the membrane were removed, and the cells on the lower surface were fixed with 4% paraformaldehyde and stained with 0.5% crystal violet solution. Images were obtained and analyzed using NIH ImageJ software (Version 4.0.4).

### Xenograft model

Five-week-old male BALB/c-nude mice were obtained from Beijing Vital River Laboratory Animal Technology Co., Ltd. All animals were housed in the Laboratory Animal Center of Soochow University and cared for in accordance with the NIH Guide for the Care and Use of Laboratory Animals. The experimental protocol was approved by the Laboratory Animal Ethics Committee of the Experimental Animal Center of Soochow University (approval no. 202011A070). Approximately 5×10^6^ normal H1299 cells or stable YTHDC2-overexpressing H1299 cells were implanted subcutaneously into the right flank of the animals (n=8 mice per group). Animals were euthanized by cervical dislocation ~30 days after implantation, and tumors were collected and photographed. The maximum xenograft tumor size obtained in the study was 12 mm.

### IHC

Tumor samples obtained from xenograft model were fixed with 4% paraformaldehyde, dehydrated through a graded series of ethanol and embedded in paraffin. Sections (3-μm thick) were deparaffinized, rehydrated, and stained with hematoxylin and eosin. For IHC, the tissue sections were blocked with 5% goat serum and incubated with primary antibodies at 4 °C overnight. Next, the sections were incubated with a goat anti-rabbit secondary antibody for 20 min at room temperature, and then subjected to streptavidin-HRP staining for 30 min. After staining with diaminobenzidine, the sections were stained with hematoxylin and subjected to dehydration. The primary antibodies used for IHC assay were purchased from Abcam (anti-YTHDC2) and ProteinTech Group, Inc. (anti-Ki67, anti-cyclin D1, anti-CDH1, anti-CDH2, anti-CYLD and anti-NF-κBp65).

### Copy number analysis

In order to explore the association between continuous *YTHDC2* mRNA expression and discrete copy number status, patients with LUAD and LUSC were distributed into 5 subgroups (homozygous deletion, single copy deletion, normal diploid copy, low-level copy number amplification and high-level copy number amplification) 41. In order to obtain the prognostic significance, the prognostic significance and the correlation between DNA copy number and smoking history were analyzed. The copy numbers of *YTHDC2* in patients with lung cancer were also analyzed using the Oncomine database (https://www.oncomine.org/resource/login.html), which is an online platform used for genome-wide data-mining 42 with the following parameters: The threshold P-value was set at 0.001 with a minimum 2-fold change. Data cohorts that had prominent copy number loss in NSCLC compared with that in normal tissue were extracted for analysis.

### TaqMan copy number assay

Total genomic DNA of CS-exposed cells and lung cancer cell lines were isolated using a genomic DNA isolation kit (Tiangen Biotech Co., Ltd.). TaqMan copy number assays for *YTHDC2* (probe ID: Hs06075146_cn) were performed according to the manufacturer's instructions. In total, 20 ng genomic DNA was mixed with 1 μl *YTHDC2* copy number assay (cat. no. 4400291; Thermo Fisher Scientific, Inc.), 1 μl copy number reference assay (cat. no. 4403326; Thermo Fisher Scientific, Inc.) and 10 μl Master Mix (cat. no. A30865; Thermo Fisher Scientific, Inc.) to a final volume of 20 μl. The reference gene in the copy number reference assay was RNase P labeled with VIC dye, which is known to have two copies in a diploid genome. Copy number PCR was performed with a QuantStudio 6 Flex Real-Time PCR system (Thermo Fisher Scientific, Inc.) and further analyzed with CopyCaller v2.1 (Thermo Fisher Scientific, Inc.) following the manufacturer's instructions.

### RNA immunoprecipitation (RIP) sequencing

RIP-RNA high-throughput sequencing was performed by Shanghai Cloud-seq Biotech Co.,Ltd. RIP was performed as previously described 43. RNA was extracted using TRIzol^®^ and ribosomal RNAs (rRNAs) were removed using NEBNext rRNA Depletion Kit (New England BioLabs, Inc.). RNA libraries were constructed using rRNA-depleted RNAs with NEBNext^®^ Ultra™ II Directional RNA Library Prep Kit (New England BioLabs, Inc.) according to the manufacturer's instructions. Libraries were controlled for quality and quantified using the BioAnalyzer 2100 system (Agilent Technologies, Inc.). Library sequencing was performed on an Illumina HiSeq instrument with 150-bp paired end reads.

### RNA decay assay

RNA decay assay was performed as previously described 44. H1299 cells transfected with pYTHDC2 or blank vector were seeded on 6-well plates, and actinomycin D (Hangzhou Fude Biological Technology Co. LTD) at a final concentration of 10 μg/ml was added to the medium. Cells were collected at 0, 1, 2, 3, 4 and 5 h after addition of actinomycin D. RNA was extracted and quantified by RT-qPCR. The same aforementioned primers were used to detect CYLD mRNA levels. Ct values were normalized to the Ct value of t = 0 to obtain ∆Ct value (∆Ct = Mean Ct of each time point - Mean Ct of t=0), and the relative abundance for each time point were calculated (mRNA abundance = 2^-∆CT^).

### Electrophoretic mobility shift assay (EMSA)

Nuclear proteins from H1299 cells were isolated using Nuclear and Cytoplasmic Protein Extraction kit (CWBio) according to the manufacturer's protocol. Protein concentration was measured using an enhanced BCA protein assay kit (Beyotime Institute of Biotechnology). Biotin-labeled NF-κB consensus oligonucleotides were obtained from Beyotime Institute of Biotechnology. Nuclear extracts (5 μg) were added to 20 μl binding reactions and incubated for 20 min at room temperature. EMSA was performed with Chemiluminescent EMSA kit (Beyotime Institute of Biotechnology) according to the manufacturer's protocol.

### m6A methylated RIP (meRIP)

The m^6^A distribution in the CYLD mRNA sequence was predicted using the sequence-based RNA adenosine methylation site predictor (SRAMP) algorithm 45. Specific primers were designed for meRIP analysis according to the high-confidence fragments. Magna MeRIP m^6^A kit (cat. no. 17-10499; MilliporeSigma) was used to analyze the m^6^A modification in H1299 cells according to the manufacturer's recommendations. In brief, 150 μg total RNA was isolated and randomly fragmented into fragments of ~100 bp. RNA samples were then immunoprecipitated with anti-m^6^A or anti-mouse IgG antibody-coated magnetic beads. The m^6^A-modified RNA fragments were eluted and analyzed by PCR and RT-qPCR. Relative enrichment of m^6^A was normalized to the input.

### Statistical analysis

GraphPad Prism v7.0 software (GraphPad Software, Inc.) was used for all statistical analyses. Values are presented as the mean ± standard deviation (n=3). Student's unpaired t-test was used to detect the statistical differences in expression of *YTHDC2* between tumor and adjacent non-tumor tissues, as well as between clinicopathological features and smoking history. Kaplan-Meier survival curves and Cox regression analysis were performed using the R survival package. Pearson's correlation coefficients were calculated to evaluate the association between DNA copy number and *YTHDC2* expression using R software (Version 4.0.4). *P*<0.05 was considered to indicate a statistically significant difference.

## Results

### Expression and clinical significance of YTHDC2 in lung cancer

**Figure 1:**
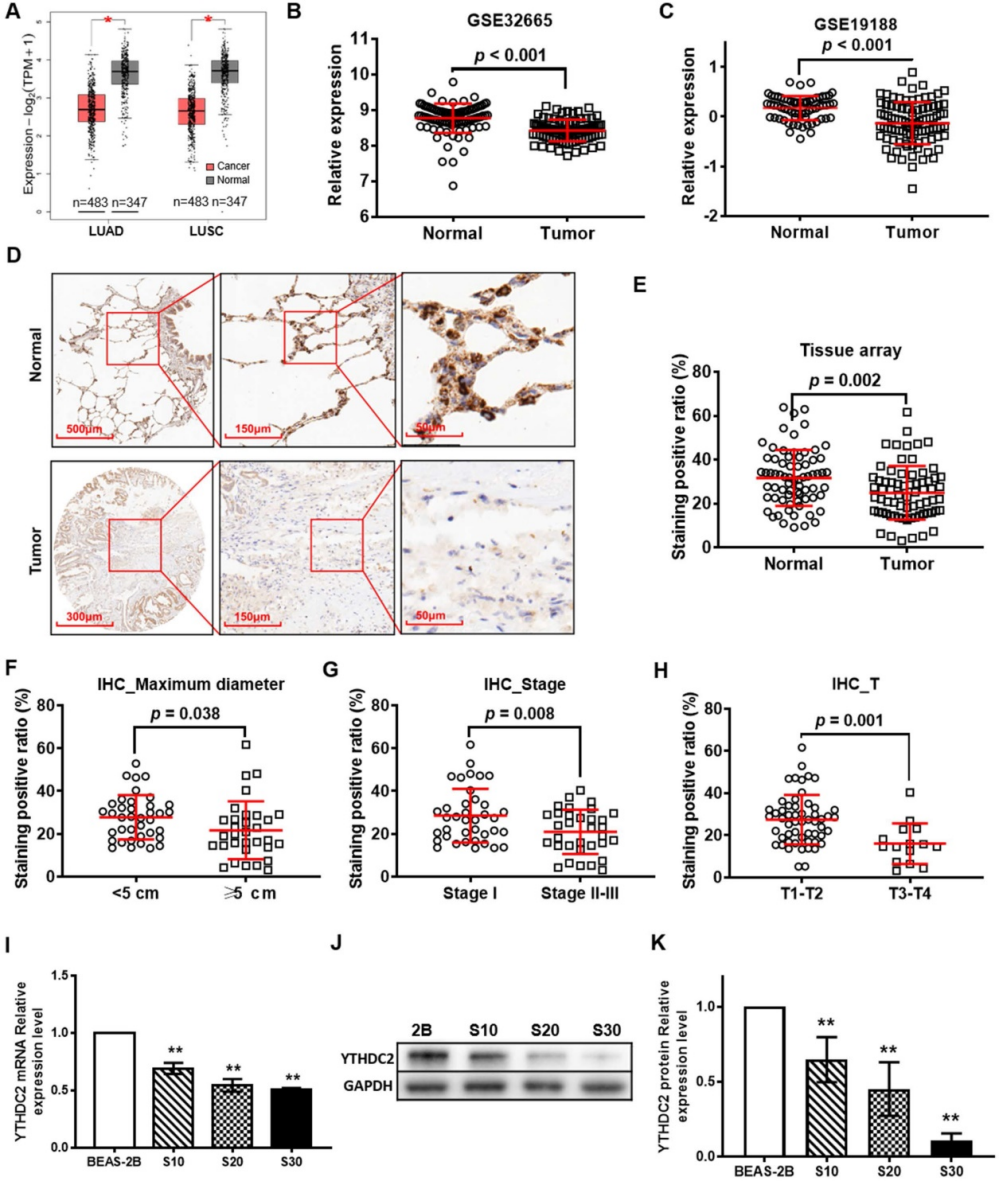
*YTHDC2* gene and protein expression is downregulated in patients with lung cancer from The Cancer Gene Atlas, Gene Expression Omnibus and Human Protein Atlas databases, as well as in CS-exposed cells. (A) Differential analysis of *YTHDC2* mRNA expression in lung cancer tissues based on the Gene Expression Profiling Interactive Analysis tool. ^*^P<0.05 vs. normal tissues. Differential analysis of YTHDC2 mRNA expression in lung cancer tissues from (B) GSE32665 and (C) GSE19188 datasets. (D) Representative IHC images showed that YTHDC2 staining was found in the cell cytoplasm in lung cancer and normal lung tissues. High expression of YTHDC2 could be found in adjacent normal tissues, while its expression was decreased in the majority of lung cancer tissues. (E) Differential analysis of YTHDC2 staining positive ratio quantitated by IHC Profiler in lung cancer tissue arrays. YTHDC2 staining positive ratio in lung cancer tissues with different (F) maximum diameter, (G) pathological stage and (H) invasion depth. (I) Relative mRNA expression level of *YTHDC2* in CS-exposed cells (S10, S20 and S30) and normal BEAS-2B cells. Western blot analysis (J) and quantitative results (K) of YTHDC2 protein expression in CS-exposed cells (S10, S20 and S30) and normal BEAS-2B cells. S10, S20 and S30 represent BEAS-2B cells exposed to CS for 10, 20 and 30 passages, respectively. **P<0.01 vs. normal BEAS-2B cells. IHC, immunohistochemistry; YTHDC2, YTH domain containing 2; CS, cigarette smoke.

According to the GEPIA online tool database, the expression levels of *YTHDC2* were observed to be different in different types of cancer. Compared with those in normal tissues, *YTHDC2* expression levels were significantly downregulated in 7 types of cancer, including LUAD and LUSC (**Figure 1A**), ovarian serous cystadenocarcinoma, testicular germ cell tumor, thyroid carcinoma, uterine corpus endometrial carcinoma and uterine carcinosarcoma, while they were increased in lymphoid neoplasm diffuse large B-cell lymphoma and thymoma (**Figure S1**). The mRNA expression of *YTHDC2* in lung cancer tissues was less than that in normal adjacent tissues in two GEO datasets, GSE32665 (**Figure 1B**) and GSE19188 (**Figure 1C**). The IHC results of lung cancer tissue arrays showed a significantly lower YTHDC2 expression in lung cancer tissues than that in normal tissues (**Figure 1D and E, S2 and S3**). Based on the data of HPA, 50% of the lung cancer tissues showed no staining for anti-YTHDC2 antibody, while the 3 normal tissues showed low staining (**Figure S4**). The present IHC results showed decreased YTHDC2 protein expression in tumor tissues with larger diameters (maximum diameter ≥5 cm), or at advanced T stages (T3 and T4) or pathological stages (**Figure 1F-H**).

When the pathological stage is considered, the expression of *YTHDC2* mRNA in stage II lung cancer tissues in the TCGA LUAD and GSE41271 cohorts was significantly lower than that in stage I (**Figure S4A and C**). The mRNA expression of *YTHDC2* in stage IV tumor tissues in the TCGA LUSC cohort was significantly lower than that in stage I (**Figure S4B**). For invasion depth T stage, the mRNA expression of *YTHDC2* in lung cancer tissues with T3 or/and T4 stages in the TCGA LUAD, TCGA LUSC and GSE30219 cohorts were also significantly lower than those in lung cancer tissues with T1 stage (**Figure S4D-F**). In addition, the expression of *YTHDC2* in M1 lung cancer was significantly lower than that in M0 lung cancer in the TCGA LUSC cohort (**Figure S4H**). Similarly, for lymph node metastasis N stage, the expression of *YTHDC2* in N1-stage lung cancer tissues in the TCGA LUAD and GSE30219 cohorts was significantly lower than that in N0 stage tissues, and the expression of *YTHDC2* in N2 stage lung cancer tissues in the GSE30219 cohort was also significantly lower than that in N0 stage tissues (**Figure S4J-L**). To identify the association between *YTHDC2* expression and smoking history, the present study analyzed the differential expression of *YTHDC2* mRNA in patients with lung cancer with different smoking histories. For the TCGA LUAD cohort, the *YTHDC2* mRNA expression level was low in current smokers and reformed smokers for ≤15 years compared with that in individuals who had never smoked. Higher *YTHDC2* mRNA expression level was found in reformed smokers for >15 years compared with that in current smokers (**Figure S4M**). *YTHDC2* mRNA expression in smoking patients was remarkedly lower than that of non-smokers in the GSE41271 cohort (**Figure S4O**).

Next, the present study evaluated whether YTHDC2 was downregulated in our established CS-induced carcinogenic model 10. RT-qPCR and western blotting revealed that *YTHDC2* was significantly downregulated in CS-exposed cells (S10, S20 and S30) at the mRNA and protein levels compared with that in unexposed BEAS-2B cells. The expression levels were found to be dependent on smoke-exposure time (**Figure 1I-K**).

As shown in **Figure S5**, univariate Cox model and Kaplan-Meier survival analysis revealed that increased *YTHDC2* mRNA expression was significantly associated with improved survival (HR<1, P<0.05) in GSE41271, GSE30219 and TCGA lung cancer datasets.

### YTHDC2 functional prediction by correlation and proteomic analyses

**Figure 2:**
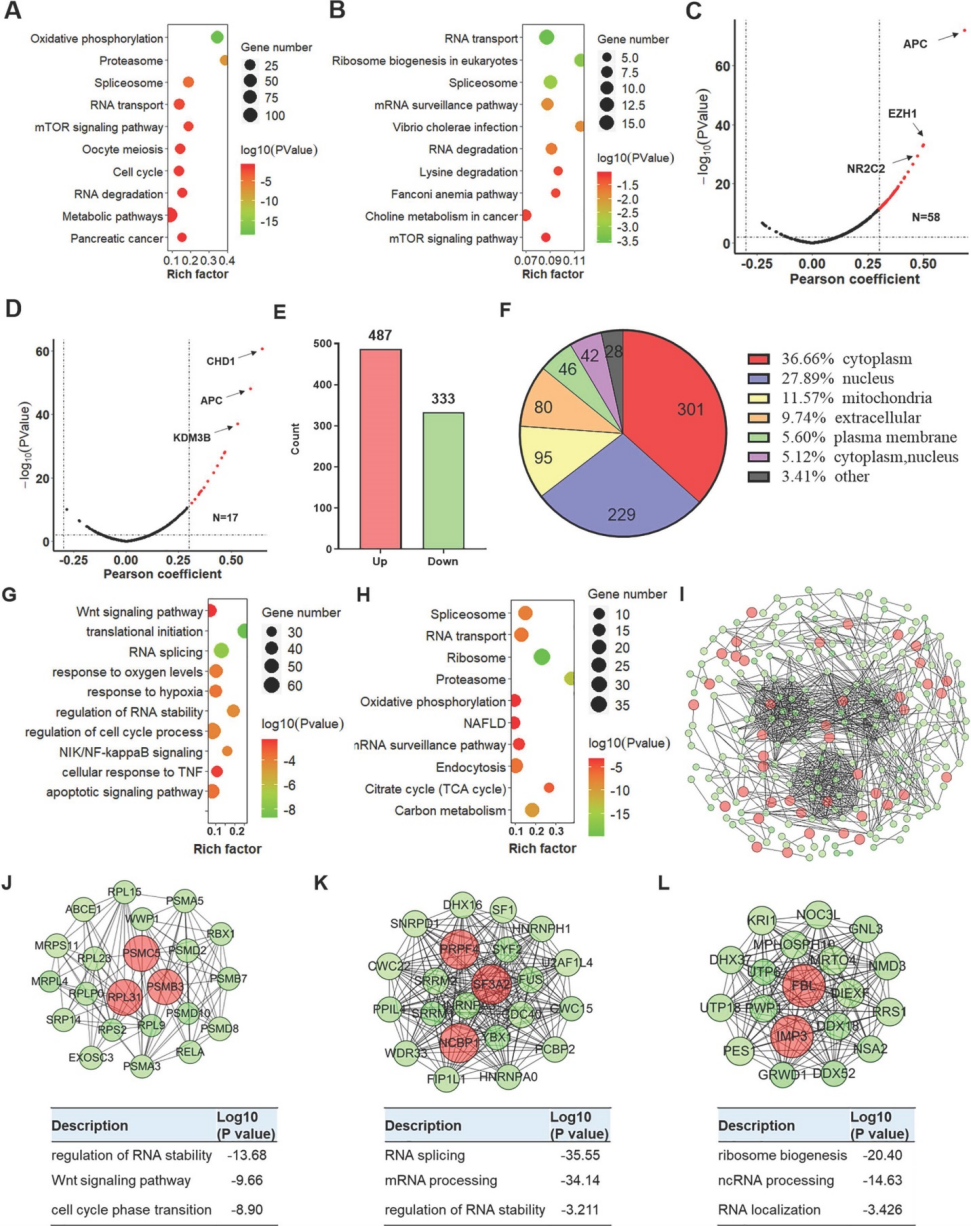
Proteomics analysis of YTHDC2-knockdown cells. Bubble chart showing the KEGG enrichment results of the genes associated with (A) LUAD and (B) LUSC. The larger the rich factor, the greater the degree of enrichment. The color gradient from red to green represents the P-value; the closer to green color, the lower the P-value and the higher the significance level corresponding to the enrichment. Volcano plots showing the tumor suppressor genes in YTHDC2-related genes in (C) LUAD and (D) LUSC. (E) Bar graph showing the number of upregulated and downregulated proteins in YTHDC2-knockdown cells. (F) Subcellular distribution of DEGs. (G) Biological process and (H) KEGG pathway analysis of DEGs. (I) The protein-protein interaction network of DEGs was constructed using Cytoscape. (J-L) Identification of the top 3 significant clusters using the plug-in Minimal Common Oncology Data Elements in Cytoscape, and biological process enrichment analysis of the proteins in these clusters. KEGG, Kyoto Encyclopedia of Genes and Genomes; YTHDC2, YTH domain containing 2; LUSC, lung squamous cell carcinoma; LUAD**,** lung adenocarcinoma; DEGs, differentially expressed genes.

Based on the analysis of the LinkedOmics online tool, 1**,**037 and 724 genes, respectively, were positively correlated with low *YTHDC2* expression [correlation coefficient (r) >0.3, P<0.001] in LUAD and LUSC, while 648 and 147 genes were negatively correlated (r <-0.3, P<0.001), respectively (**Figure S6A-B**). Further Kyoto Encyclopedia of Genes and Genomes **(**KEGG**)** enrichment analysis showed that these *YTHDC2*-related genes in LUAD were significantly enriched in **'**RNA transport**'**, **'**mTOR signaling pathway**'**, **'**cell cycle**'** and** '**RNA degradation**'** (**Figure 2A**), while the *YTHDC2***-**related genes in LUSC were significantly enriched in; RNA transport**'** and **'**RNA degradation**'** (**Figure 2B**). For biological process enrichment, the *YTHDC2***-**related genes in LUAD and LUSC were also enriched in several cancer-related processes, including 'NF-κB signaling', 'mRNA splicing', 'cell cycle', 'cell division', 'TNF-mediated signaling**'** and **'**Wnt signaling pathway**'** (**Figure S6C-D**). TSGs and MRGs were obtained from the TSGene and HCMDB databases. A total of 58 and 17 TSGs was positively associated with *YTHDC2* in LUAD (**Figure 2C**) and LUSC (**Figure 2D**), respectively. A total of 62 and 12 MRGs were negatively correlated with *YTHDC2* in LUAD (**Figure S6I**) and LUSC (**Figure S6J**), while 89 and 31 MRGs were positively correlated with *YTHDC2* in LUAD (**Figure S6I**) and LUSC (**Figure S6J**), respectively. Among these positively correlated TSGs and MRGs, adenomatous polyposis coli**,** chromodomain helicase DNA binding protein 1 and enhancer of zeste homolog 2 were found to be significantly reduced in CS-exposed cells (S10, S20 and S30) and lung cancer cells (A549 and H1299) (**Figure S6K-M**).

In addition, among the quantified differentially expressed proteins (DEPs) identified by TMT proteomics profiling, 487 were upregulated and 333 were downregulated (**Figure 2E and Table S1**). Based on the subcellular location annotation, certain DEPs were found ≥1 locations within the cell: In total, 36.66% were in the cytoplasm, 27.89% in the nucleus, 11.57% in mitochondria, 9.74% in the extracellular matrix and 5.60% in the plasma membrane (**Figure 2F**). To assess the potential biological function of YTHDC2, DEPs were subjected to GO functional and KEGG pathway enrichment. According to biological process classification, these DEPs were involved in various cellular processes, including 'translational initiation', 'RNA splicing', 'response to hypoxia', 'regulation of RNA stability' and 'cell cycle process and apoptotic signaling' (**Figure 2G**). Regarding KEGG pathway enrichment, these DEPs were enriched in several important pathways, including 'spliceosome', 'ribosome', 'proteasome', 'citrate cycle' and 'endocytosis' (**Figure 2H**). Among the 820 DEPs, 264 proteins were depicted in the STRING database to identify several clusters by plug-in Molecular Complex Detection (MCODE) in Cytoscape (**Figure 2I**). The DEPs in the top 3 clusters were imported into the DAVID database for biological process enrichment. The cluster 1 sub-network was related to 'RNA splicing', 'mRNA processing' and 'regulation of RNA stability'; cluster 2 sub-network was linked to 'non-coding RNA (ncRNA) localization', 'RNA localization' and 'ribosome biogenesis'; and cluster 3 was involved in the 'regulation of RNA stability', 'Wnt signaling pathway' and 'cell cycle phase transition' (**Figure 2J-K**).

### YTHDC2 downregulation increases the proliferation and migration of lung cancer cells

**Figure 3:**
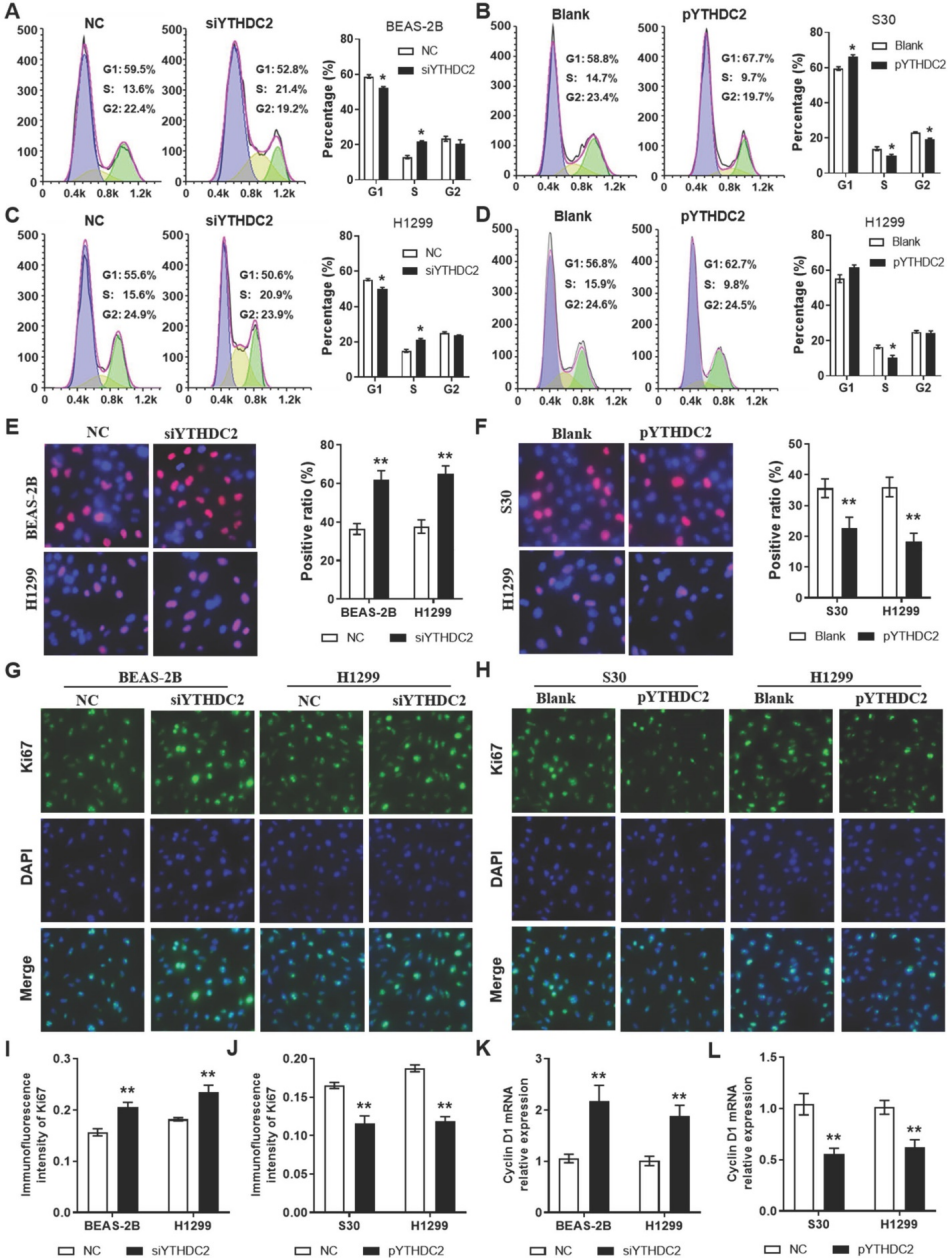
*YTHDC2* downregulation promotes lung cancer cell proliferation. Representative images and quantification results of cell cycle of (A) BEAS-2B and (C) H1299 cells transfected with siYTHDC2 and NC. Representative images and quantification results of cell cycle of (B) S30 and (D) H1299 cells transfected with overexpression vector (pYTHDC2) and blank vector. (E) Cell proliferation was measured in BEAS-2B and H1299 cells transfected with siYTHDC2 and NC by EdU cell proliferation assay. (F) Cell proliferation were evaluated in S30 and H1299 cells transfected with pYTHDC2 and blank control by EdU cell proliferation assay. (G) Representative immunofluorescence images and (I) quantification results of Ki67 in BEAS-2B and H1299 cells transfected with siYTHDC2 and NC. (H) Representative immunofluorescence images and (J) quantification results of Ki67 in S30 and H1299 cells transfected with pYTHDC2 and blank control. Relative mRNA expression level of cyclin D1 in (K) BEAS-2B and H1299 cells transfected with siYTHDC2 and NC, as well as in (L) S30 and H1299 cells transfected with pYTHDC2 and blank control. ^*^P<0.05 vs. NC or blank control group, ^**^P<0.01 vs. NC or blank control group. YTHDC2, YTH domain containing 2; siRNA, small interfering RNA; NC, negative control; EdU, 5-ethynyl-2'-deoxyuridine.

In order to study the regulation of the *YTHDC2* gene in cancer cell proliferation, siRNA (siYTHDC2) or expression plasmid vector (pYTHDC2) was transfected into normal human lung BEAS-2B cells, as well as in the lung cancer cell line H1299 to knockdown or overexpress *YTHDC2*. Data from flow cytometry analys**i**s showed that knocking down *YTHDC2* promoted cell cycle progression by accelerating the progression of cells from the G_1_ to the S phase in BEAS-2B and H1299 cells (**Figure 3A, C**). EdU assay showed that the proliferation of BEAS-2B and H1299 cells was significantly enhanced by knocking down *YTHDC2* (**Figure 3E**). By contrast, the cell cycle progression and proliferation capacity of S30 and H1299 cells were markedly decreased by the overexpression of *YTHDC2* (**Figure 3B, D, F**). The immunofluorescence results showed an elevated Ki67 expression in YTHDC2**-**knockdown cells and a reduced expression in YTHDC2**-**overexpressing cells (**Figure 3G-J**). RT-qPCR analysis revealed the upregulation of **c**yclin D1 expression, a protein required for cell cycle G_1_/S transition, in YTHDC2-knockdown cells and a downregulation in YTHDC2-overexpressing cells (**Figure 3K-L**). These observations suggested that *YTHDC2* is required for the cell cycle progression and proliferation of lung cancer cells.

**Figure 4:**
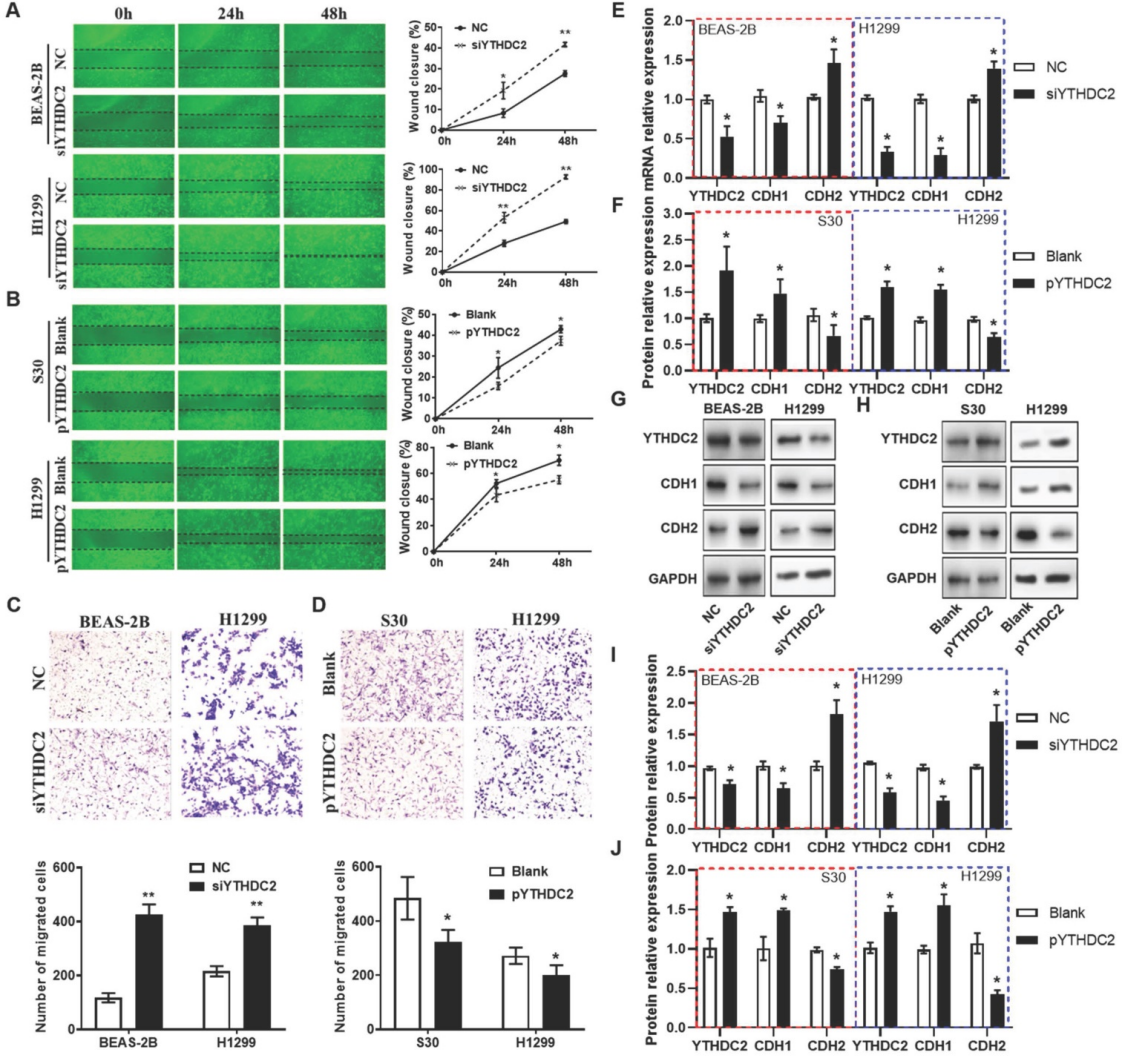
YTHDC2 downregulation promotes lung cancer cell migration. (A) Representative images and quantification of the wound healing assay showing that cell migration was significantly increased at 24 and 48 h after transfection with siYTHDC2 in BEAS-2B and H1299 cells, as well as following transfection with (B) the overexpressing vector pYTHDC2 and a blank vector. Representative images and quantification of the Transwell migration assay of BEAS-2B and H1299 cells transfected with (C) siYTHDC2 and (D) pYTHDC2. (E) Quantitative PCR analysis of CDH1 and CDH2 in normal BEAS-2B andH1299 cells transfected with siYTHDC2, (F) as well as in S30 and H1299 cells transfected with pYTHDC2. (G) Western blot analysis and (I) quantitative results of EMT markers in BEAS-2B and H1299 cells transfected with siYTHDC2. (H) Western blot analysis and (J) quantitative results of EMT markers in S30 and H1299 cells transfected with pYTHDC2. *P<0.05 vs. NC (blank vector) group, **P<0.01 vs. NC (blank vector) group. YTHDC2, YTH domain containing 2; siRNA, small interfering RNA; EMT, epithelial-mesenchymal transition; NC: negative control; CDH1: E-cadherin; CDH2: N-cadherin.

Regarding cell migration ability, wound healing assays (**Figure 4A and B**) and Transwell assays (**Figure 4C**) also showed that the migration capacity of BEAS-2B and H1299 cells was significantly enhanced by knocking down YTHDC2. The results from RT-qPCR and western blotting revealed that the mRNA and protein levels of the mesenchymal marker N-cadherin (*CDH2*) were significantly upregulated, while the epithelial marker E-cadherin (*CDH1*) was downregulated in siYTHDC2-transfected cells compared with the findings in the negative control group (**Figure 4E**). By contrast, *CDH2* was significantly downregulated, while *CDH1* was upregulated, in pYTHDC2-transfected cells compared with the findings in the blank vector group (**Figure 4F**). In addition, the changes in CDH1 and CDH2 protein levels were consistent with the mRNA levels in both knockdown and overexpressing cells (**Figure 4G-J**). These observations suggested that *YTHDC2* is required for the cellular migration and EMT process of lung cancer cells.

### YTHDC2 upregulation suppresses lung cancer cell tumorigenesis *in vivo*

**Figure 5:**
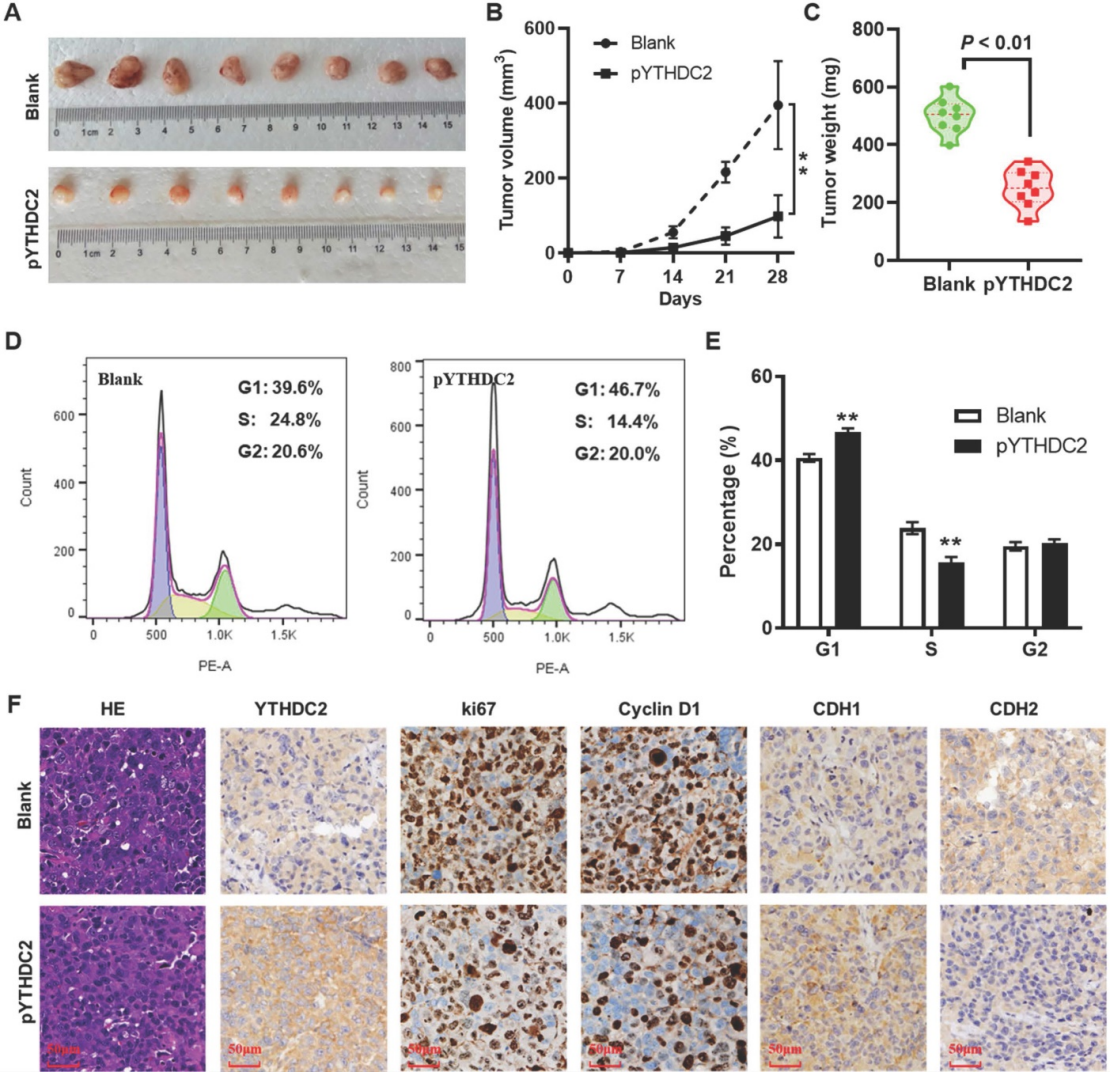
YTHDC2 overexpression suppresses H1299 cells growth* in vivo*. (A) Images of the xenograft tumors formed in nude mice injected with YTHDC2-overexpressing and control cells. (B) Volume and (C) weight of xenograft tumors isolated from nude mice. (D) Representative images and (E) quantification of the results of cell cycle analysis of single cell suspensions yielded from xenograft tumors. (F) Representative images of hematoxylin and eosin staining, and immunohistochemical staining of YTHDC2, Ki-67, cyclin D1, E-cadherin and N-cadherin in xenograft tumors derived from nude mice. Scale bar, 50 µm; **P<0.01 vs. blank group. YTHDC2, YTH domain containing 2.

To further validate the effect of YTHDC2 on lung cancer cell tumorigenesis *in vivo*, a xenograft animal model was established by subcutaneously injecting YTHDC2-overexpressing cells into the right armpits of BALB/c nude mice. As expected, YTHDC2 overexpression markedly attenuated the growth of tumors in nude mice compared with the findings in the blank group (**Figure 5A and B**). Similarly, the mean weight of the xenograft tumors in the pYTHDC2 group was also markedly lower than that in the blank group (**Figure 5C**). To further confirm the inhibitory effect of YTHDC2 on cell proliferation that was found *in vitro*, tumor tissues were digested with trypsin to form a single-cell suspension, and the cell cycle was then detected by flow cytometry. The results suggested a stronger cell proliferation ability in the pYTHDC2 group than in the blank group (**Figure 5D and E**). In addition, a significantly lower proportion of Ki67 and cyclin D1-positive cells in xenograft tumors derived from the pYTHDC2 group compared with that of the blank group was observed by IHC analysis (**Figure 5F**). The IHC results indicated that CDH1 was upregulated, while CDH2 was downregulated, in the xenograft tumors of the pYTHDC2 group compared with the findings in the blank group (**Figure 5F**).

### Reduced YTHDC2 expression is associated with gene copy loss in DNA

**Figure 6:**
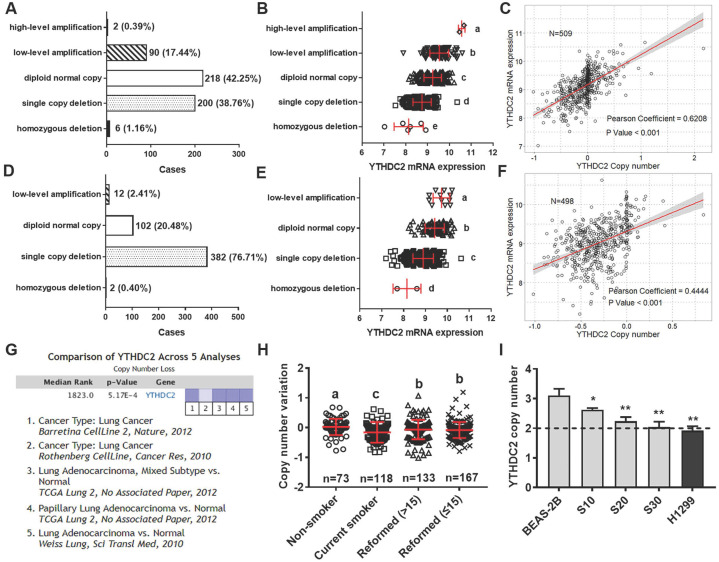
*YTHDC2* mRNA expression was regulated by gene amplification. Distribution of patients with (A) LUAD and (D) LUSC with different *YTHDC2* amplification status. *YTHDC2* mRNA expression in (B) LUAD and (E) LUSC tissues with different *YTHDC2* amplification status. Different letters (a, b, c and d) represent statistically significant group differences. Pearson's correlation analysis revealed a significant positive correlation between *YTHDC2* mRNA expression and copy numbers in (C) LUAD and (F) LUSC. The line represents linear regression of data (LUAD: y=1.065x+9.177, R^2^=0.385; LUSC: y=0.965x+9.318, R^2^=0.198). (G) The Oncomine datasets for the corresponding *YTHDC2* copy numbers in lung cancer were obtained with a threshold P=0.001 and ≥2 fold-change. The data in the graphic show significant downregulation (blue column) of *YTHDC2* copy numbers in lung cancer versus normal tissue. The intensity of the blue color represents the respective levels of *YTHDC2* copy number. (H) Copy number variation in LUAD samples with different smoking histories. Different letters (a, b, c and d) represent statistically significant group differences. (I) Copy number variation of *YTHDC2* in BEAS-2B cells and cigarette smoke-exposed cells (grey block), as well as in two lung cancer cell lines (black block). The dotted line (copy number = 2) represents the copy number of the reference gene RNase P. ^*^P<0.05, ^**^P<0.01 vs. BEAS-2B cells. YTHDC2, YTH domain containing 2; LUSC, lung squamous cell carcinoma; LUAD**,** lung adenocarcinoma.

The present study further explored the mechanisms of YTHDC2 dysregulation using the multi-omics data of lung cancer from TCGA database. A total of 509 patients with LUAD and 498 patients with LUSC had DNA amplification and mRNA expression measured simultaneously, and 39.92% of LUAD samples and 77.11% of LUSC samples showed low-level amplification (**Figure 6A and D**). Significant differences were observed in different DNA amplification groups in both lung cancer subtypes (**Figure 6B and E**). Further correlation analysis indicated that YTHDC2 mRNA expression values were significantly correlated with copy number values in LUAD and LUSC (Pearson correlation coefficient = 0.62 and 0.44, respectively; **Figure 6C and F**). In addition, 5 Oncomine datasets were extracted to verify the YTHDC2 copy numbers in lung cancer, and a significantly decreased copy number was found (**Figure 6G**).

To verify the association between *YTHDC2* copy number and smoking status, the present study analyzed the *YTHDC2* genetic amplification in patients with lung cancer with different smoking histories. Compared with that of individuals who never smoked, the *YTHDC2* copy number was significantly lower in current smokers and reformed smokers. Furthermore, the copy number was markedly increased in reformed smokers compared with that of current smokers, but it was still significantly lower than that of lifelong non-smokers (**Figure 6H**). The copy number variation of *YTHDC2* was further verified in CS-exposed BEAS-2B cells and several lung cancer cell lines using TaqMan qPCR. The *YTHDC2* copy numbers in CS-exposed cells (S10, S20 and S30) and the NSCLC cell line (H1299) was lower than that in normal cells (**Figure 6I**). However, Kaplan-Meier survival and pathological analyses showed no significant prognostic effect in either LUAD or LUSC (**Figure S8**). Furthermore, no correlation was found between *YTHDC2* copy numbers and pathological features (**Table S3**).

### CYLD is a downstream target of YTHDC2

**Figure 7:**
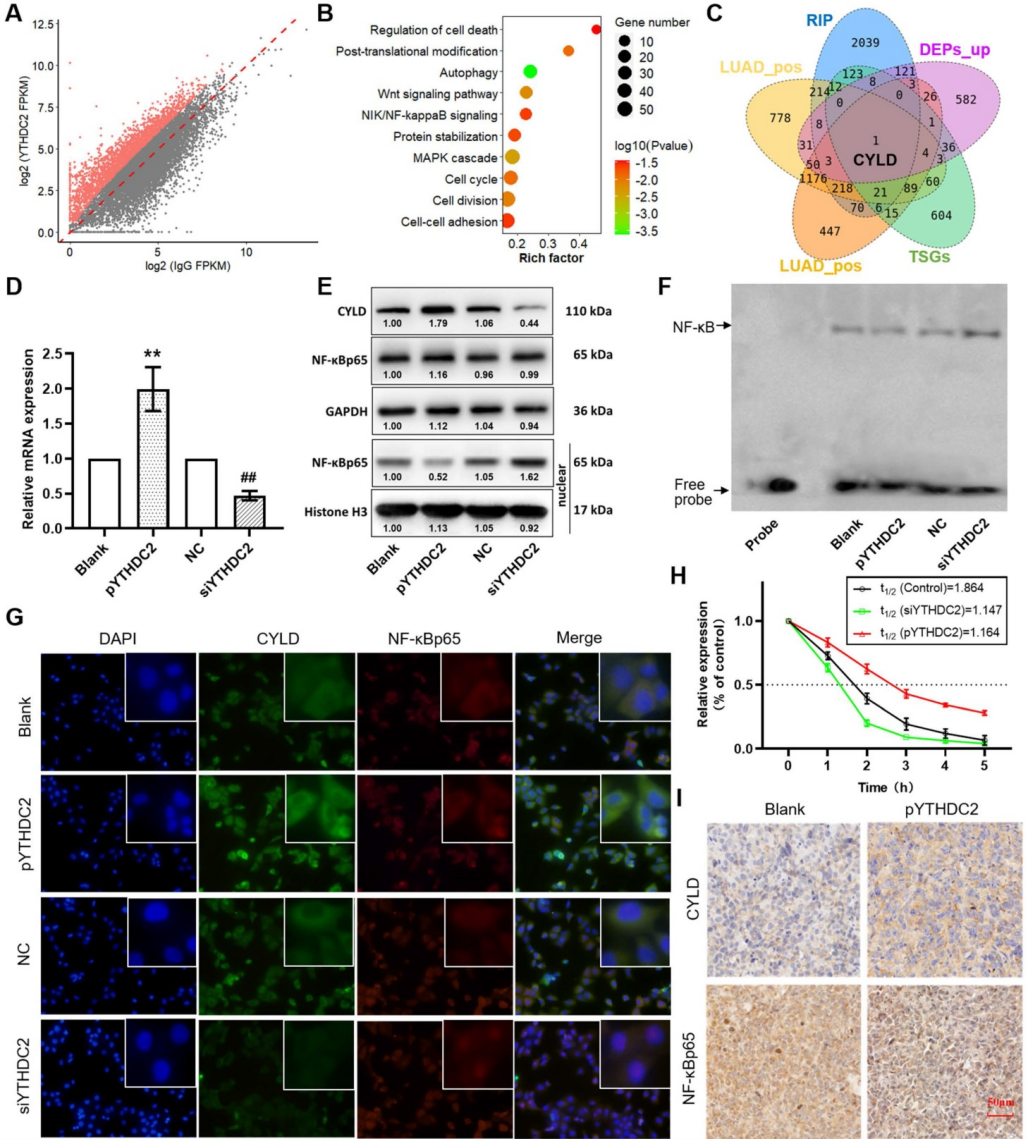
YTHDC2 promotes *CYLD* mRNA stability and inhibits NF-κB activity. (A) Scatter plot showing the mRNA transcripts identified by YTHDC2 RIP-sequencing, and transcripts that were significantly enriched are marked in red. (B) Kyoto Encyclopedia of Genes and Genomes pathway enrichment analysis of the mRNAs significantly enriched by YTHDC2 RIP. (C) Venn diagram showing the intersection of the YTHDC2 RIP-enriched mRNAs, YTHDC2-related mRNAs, upregulated differentially expressed proteins and tumor suppressor genes. (D) Relative mRNA expression level of *CYLD* in YTHDC2-overexpressing and knocked down H1299 cells. ^**^P<0.01 vs. blank group, ^##^P<0.01 vs. negative control group. (E) Relative protein expression level of CYLD in YTHDC2-overexpressing and knocked down H1299 cells. (F) RNA decay assay for *CYLD* mRNA stability upon YTHDC2 overexpression and knockdown in H1299 cells. (G) Immunostaining analysis of NF-κB p65 and CYLD in YTHDC2-overexpressing and -knockdown H1299 cells. (H) The DNA-binding activity of NF-κB in YTHDC2-overexpressing and -knockdown H1299 cells was measured by electrophoretic mobility shift assay using the biotin-labeled consensus NF-κB-binding sequence. (I) Immunohistochemistry staining of NF-κB p65 and CYLD in xenograft tumors derived from nude mice. RIP, RNA immunoprecipitation; YTHDC2, YTH domain containing 2; CYLD, cylindromatosis.

To further identify the downstream regulatory pathways of YTHDC2, RIP sequencing was performed to obtain the mRNAs that YTHDC2 can directly combine with. A total of 2,850 mRNAs were screened that were considered capable of binding to YTHDC2 under the criteria of P<0.05 and log2 (fold-change) >1 (**Figure 7A**). Enrichment analysis showed that these mRNAs were significantly enriched in several important signaling pathways, including the 'Wnt signaling pathway', 'NF-κB-inducing kinase/NF-κB signaling', 'cell cycle' and 'cell-cell adhesion' (**Figure 7B**). Notably, in the intersection of YTHDC2 RIP-enriched mRNAs, YTHDC2-related mRNAs, upregulated DEPs and TSGs, only one gene named *CYLD* was obtained (**Figure 7C**). RIP PCR assay verified the combination of YTHDC2 and *CYLD* transcript (**Figure S7C**). According to correlation analysis, the *CYLD* mRNA expression level was significantly correlated with YTHDC2 in both LUAD and LUSC (**Figure S7A and B**). Consistent with *YTHDC2*, the *CYLD* mRNA expression in lung cancer tissues was lower than that in normal adjacent tissues in GSE32665 (**Figure S9A**) and GSE19188 (**Figure S9B**), as well as in GEPIA online tool (**Figure S9C**). Moreover, the Kaplan-Meier survival analysis revealed that lung cancer patients with high *CYLD* expression have the better prognosis (**Figure S9D**). When considering smoking history, *CYLD* mRNA expression was significantly lower in tumor tissues from smokers than from non-smokers (**Figure S9E and F**). In addition, *CYLD* was significantly downregulated in CS-exposed cells (S10, S20 and S30) at the mRNA and protein levels compared with the expression levels in unexposed BEAS-2B cells (**Figure S9G-I**).

RT-qPCR and western blot analyses showed a significant upregulation of CYLD expression in YTHDC2-overexpressing H1299 cells but not in YTHDC2-knockdown cells (**Figure 7D and E**). In addition, CYLD was identified as a negative regulator of NF-κB signaling 46. The results of western blotting and immunofluorescence analyses suggested that NF-κB p65 was significantly accumulated in the nucleus, but was unchanged in total cells (**Figure 7E and G**). In addition, the results of EMSA assay suggested decreased NF-κB activity in YTHDC2-overexpressing H1299 cells, while NF-κB activity was increased in YTHDC2-knockdown cells (**Figure 7F**). The RNA decay assay demonstrated that, compared with the findings in the blank control, YTHDC2 overexpression significantly slowed down the degradation of CYLD mRNA, whereas YTHDC2 knockdown significantly promoted its degradation (**Figure 7H**). In xenograft tumors, CYLD was upregulated, while NF-κB p65 was downregulated in the pYTHDC2 group compared with the findings in the blank group (**Figure 7I**).

### YTHDC2 regulates CYLD through m^6^A modification

**Figure 8:**
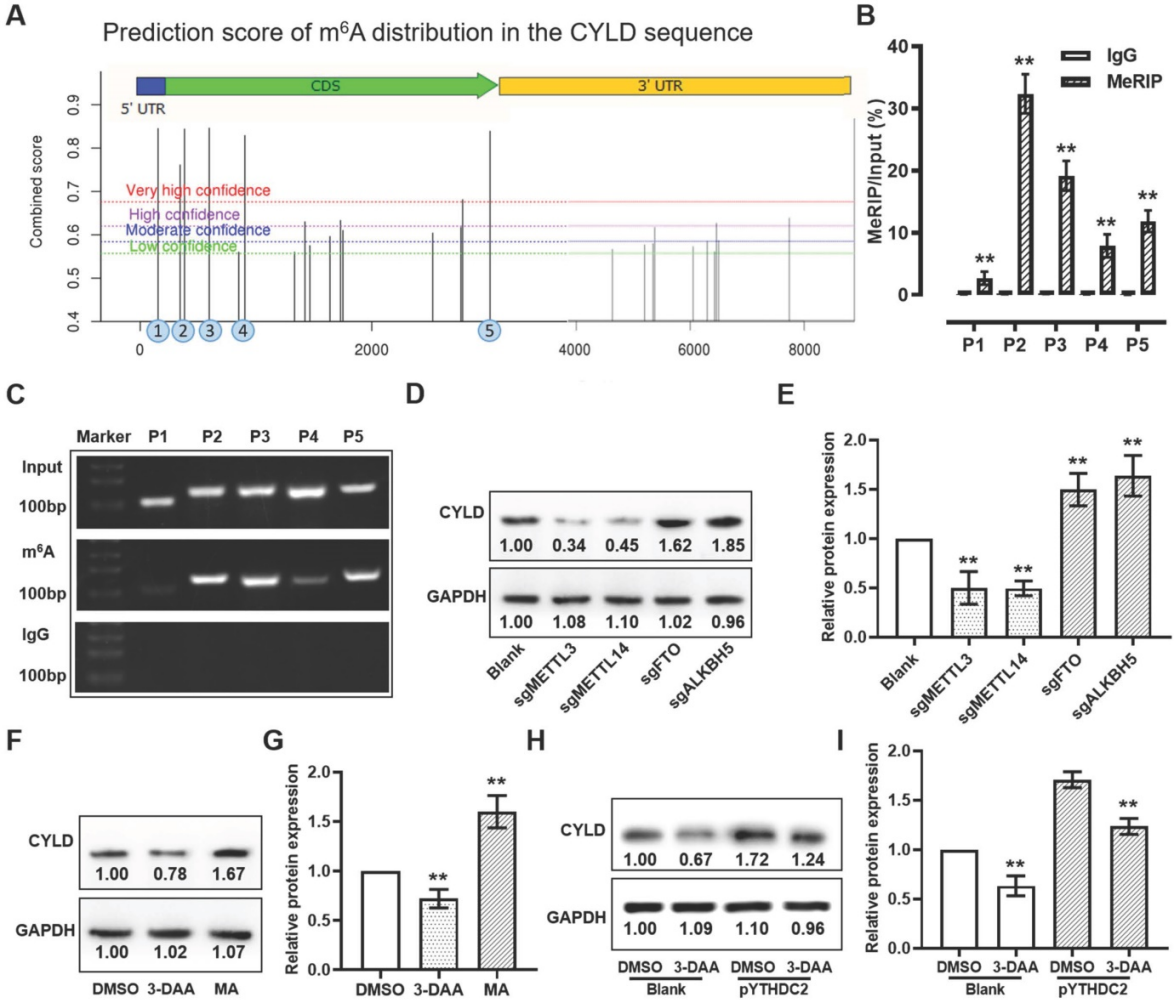
YTHDC2 regulates the stability of *CYLD* through m^6^A modification. (A) Prediction score of m^6^A distribution in CYLD sequence according to the sequence-based RNA adenosine methylation site predictor online tool. (B) meRIP-quantitative PCR and (C) meRIP-PCR results showed amplification of sites 2 to 5, indicating m^6^A modification in these four segments. Anti-IgG antibody was used as control. (D and E) Western blot results showing the relative protein expression level of CYLD in *METTL3*, *METTL14*, *FTO* and *ALKBH5* knocked down H1299 cells. (F and G) Western blot result showing the relative protein expression level of CYLD in H1299 cells treated with DAA, a global methylation inhibitor, and with meclofenamic acid, a FTO inhibitor. (H and I) Western blot results showing the relative protein expression level of CYLD in YTHDC2-overexpressing H1299 cells treated with or without 3-DAA. YTHDC2, YTH domain containing 2; CYLD, cylindromatosis; meRIP, m^6^A methylated RNA immunoprecipitation; m^6^A, N6-methyladenosine; METTL, methyltransferase-like; FTO, fat mass and obesity-associated protein; DAA, 3-deazaadenosine.

According to the SRAMP online tool, 4 predicted m^6^A methylated sites with markedly high confidence was found among the CYLD mRNA sequence (Fi**gure 8A**). To validate these m^6^A sites, meRIP assay was used to obtain the methylated RNA, followed by qPCR and PCR. The qPCR and PCR results suggested that, after normalization of m^6^A signals of specific segment to input, 4 m^6^A sites (sites 2-5) from the 5 predicted regions with markedly high confidence showed more elevated levels, suggesting that the m^6^A modification was present in the sequence of these sites (**Figure 8B and C**). To further verify whether CYLD mRNA was regulated by m^6^A modification, global m^6^A methylation was modified by knocking down the m^6^A methylases METTL3/14 and demethylases FTO/ALKBH5, and by treatment with the global methylation inhibitor 3-deazaadenosine (DAA) and the FTO inhibitor meclofenamic acid (MA). The results demonstrated that, when decreasing the m^6^A level by knocking down METTL3/14 or by treatment with 3-DAA, the relative protein expression level of CYLD showed a significant decrease. Instead, when increasing the m^6^A level by knocking down FTO/ALKBH5 or by treatment with MA, the CYLD relative protein expression level was markedly upregulated (**Figure 8D-G**). Importantly, the upregulated CYLD protein expression triggered by YTHDC2 overexpression could be significantly reduced by 3-DAA treatment, suggesting that YTHDC2 regulates CYLD stability through m^6^A modification (**Figure 8H and I**).

## Discussion

Members of the YTH domain family of proteins serve as crucial regulators of gene expression by specifically recognizing and binding to RNAs containing m^6^A 47. YTHDC1 has been shown to play an important regulatory role in pre-mRNA splicing in the nucleus, and YTHDF1, YTHDF2 and YTHDF3 have been found to play a synergistic role in promoting the effective translation and degradation of specific m^6^A-containing RNA in the cytoplasm 17, 18. It is noteworthy that YTHDC2, the fifth member of the YTH protein family, exists both in the cytoplasm and in the nucleus. It has been demonstrated that YTHDC2 can modulate the expression and degradation of specific mRNAs in different types of cells 47. *YTHDC2* has been recognized to be associated with the development of certain types of cancer, including HCC 27 and colon cancer 26. The present study used the GEPIA online tool to analyze the expression of *YTHDC2,* and found it to be significantly decreased in several solid tumors, including the two most common subtypes of lung cancer, compared with its expression in normal tissues.

The present study demonstrated that the expression of YTHDC2 was significantly downregulated in CS-exposed BEAS-2B cells. Based on bioinformatics and tissue array IHC analyses, the mRNA and protein expression of YTHDC2 was downregulated in lung cancer tissues compared with that of normal tissues. It was found that the expression of YTHDC2 was significantly lower in smoking patients than in non-smoking patients, which was consistent with the present *in vitro* experiments, suggesting that smoking could reduce the expression of YTHDC2. These results suggested that YTHDC2 is an important smoking-related gene in the regulation of the malignant potential of lung cancer cells. The current results showed different YTHDC2 expression profiles and prognostic significance in patients with lung cancer. However, the precise signaling pathways involved in the biological processes still need to be defined. *YTHDC2* has been reported to contribute to colon cancer metastasis by promoting the translation of HIF-1α and its related pathways. The DEPs in YTHDC2-knockdown cells were linked to translation initiation, RNA splicing and RNA stability, which is consistent with the previously reported role of YTHDC2 in transcription 47. The present results also showed that these DEPs are correlated with hypoxia, which occurs pathologically in solid tumors, and induces angiogenesis and tumor cell migration 48. In addition, the bioinformatics and proteomics analyses demonstrated that YTHDC2 contributed to the initiation and progression of lung cancer. Decreased *YTHDC2* expression in normal BEAS-2B lung cells and H1299 LUAD cells significantly enhanced cell migration. When *YTHDC2* was knocked down in BEAS-2B cells, the expression of the epithelial marker *CDH1* was significantly downregulated, while the expression of the mesenchymal marker *CDH2* was significantly upregulated. Importantly, the xenograft animal model further validated the tumor-suppressor effect of YTHDC2 on lung cancer cell tumorigenesis *in vivo*. These observations suggested that decreased expression of *YTHDC2* contributes to enhanced EMT ability, which is closely associated with the metastasis and progression of tumors.

Variation in gene copy number of >50 bp in length is generally considered to play an essential role in the development of human tumors 49. The loss of TSGs and the gain of proto-oncogenes were shown to contribute to cancer development 50, 51. It has been reported that the copy numbers and expression levels of programmed death-ligand 1 were both increased in smoking-related NSCLC 52. Another study revealed that there was a significant correlation between the expression and copy number variation of EMT-related genes in numerous tumor samples of different cancer types 53. In the present study, *YTHDC2* expression was significantly correlated with copy number, and a decreased *YTHDC2* copy number was highly associated with patient smoking history. Nevertheless, increased *YTHDC2* copy number had no prognostic value in patients with lung cancer, since there was no significant correlation between *YTHDC2* copy number and pathological features.

CYLD was reported as a tumor suppressor that participates in the occurrence and development of multiple cancer types, including pancreatic cancer 54, colon carcinoma 55, breast cancer 56 and HCC 57. CYLD is a deubiquitinating enzyme that inhibits polyubiquitin-dependent pathways, including the NF-κB signaling pathway, which can promote cell growth and inflammatory 58. However, as an important TSG, the upstream regulatory mechanism of CYLD in cancer remains unclear. A previous study reported that CYLD can be regulated by lncRNA CRAL/miR-505 in human gastric cancer cells to reverse cisplatin resistance 59. Several other miRNAs were also reported to regulate CYLD in multiple cancer types. For example, in colon cancer, miR-181b was able to target CYLD to inhibit the NF-κB signaling pathway 60, while, in cervical cancer, miR-501 can target CYLD to regulate cell proliferation, migration and invasion 61. The present study identified multiple m^6^A methylation sites in CYLD transcripts from an epigenetics perspective, which were recognized and bound by the m^6^A reader YTHDC2, thus further contributing to CYLD mRNA stability. YTH domain-containing readers have been reported to be able to regulate the splicing, translation or stability of specific m^6^A modified mRNAs 47. A previous study demonstrated that YTHDC2 can suppress LUAD tumorigenesis via promoting solute carrier family 7 member 11 (SLC7A11) decay 62. The current study identified CYLD as a target of YTHDC2 based on bioinformatics analysis, RIP sequencing and proteomic analysis, and found that YTHDC2 is able to promote CYLD mRNA stability.

In conclusion, the present results indicated that the downregulation of smoking-related YTHDC2 was associated with enhanced proliferation and migration of lung cancer cells, and appeared to be regulated by DNA copy number variation. CYLD/NF-κB is a vital downstream pathway that contributes to the tumor-suppressor role of YTHDC2 in lung cancer cells. Further *in vitro* and* in vivo* studies are needed to gain insights into additional m^6^A-dependent downstream mechanisms of YTHDC2 in lung cancer.

## Supplementary Material

Supplementary figures and tables.

## Abbreviations

SCLC: small cell lung cancer
NSCLC: non-small cell lung cancer
LUSC: lung squamous cell carcinoma
LUAD: lung adenocarcinoma
OS: overall survival
HCC: hepatocellular carcinoma
GEO: Gene Expression Omnibus
TCGA: The Cancer Gene Atlas
UCSC: University of California Santa Cruz
RSEM: RNA-seq expression value
HRs: hazard ratios
CIs: confidence intervals
TSGs: tumor suppressor genes
MRGs: metastasis-related genes
cDNA: complementary DNA
ATCC: American Type Culture Collection
FBS: fetal bovine serum
CDS: coding sequence
SD: standard deviation
HPA: Human Protein Atlas
EMT: epithelial-mesenchymal transformation
CDH1: E-cadherin
CDH2: N-Cadherin
CNV: copy number variation
RIP: RNA immunoprecipitation
meRIP: m^6^A methylated RNA immunoprecipitation
EMSA: Electrophoretic mobility shift assay
