# Supplementary material for: Downregulation of m6A Reader YTHDC2 Promotes the Proliferation and Migration of Malignant Lung Cells via CYLD/NF-κB Pathway
Source: Int J Biol Sci. 2021 Jun 22;17(10):2633–51. doi: 10.7150/ijbs.58514 (PMC8315025; doi:10.7150/ijbs.58514)
Supplement: Supplementary file 1 — Supplementary figures and tables. [file ijbsv17p2633s1.pdf]

# *Supplementary Material*

## 1. Supplementary Tables

**Table S1** Characterization of datasets used in this study

| Dataset | Normal | NSCLC | Stage | T | N | M | Smoking | Survival data |
|---|---|---|---|---|---|---|---|---|
| TCGA_LUAD | 59 | 515 | Y | Y | Y | Y | Y | Y |
| TCGA_LUSC | 51 | 502 | Y | Y | Y | Y | Y | Y |
| GSE41271 | 0 | 275 | Y | N | N | N | Y | Y |
| GSE30219 | 0 | 293 | N | Y | Y | Y | N | Y |
| GSE32665 | 92 | 87 | N | N | N | N | N | N |
| GSE19188 | 65 | 91 | N | N | N | N | N | N |

LUAD: lung adenocarcinoma; LUSC: Squamous cell carcinoma; TCGA: The Cancer Gene Atlas; T: Invasion depth; N: Lymph node metastasis; M: Distant metastasis

**Table S2** Primers used in this study

| Primer name | Primer sequence 5′ to 3′ |
|---|---|
| F_YTHDC2 | CAAAACATGCTGTTAGGAGCCT |
| R_YTHDC2 | CCACTTGTCTTGCTCATTTCCC |
| F_CDH1 | CGAGAGCTACACGTTCACGG |
| R_CDH1 | GGGTGTCGAGGGAAAAATAGG |
| F_CDH2 | AGCCAACCTTAACTGAGGAGT |
| R_CDH2 | GGCAAGTTGATTGGAGGGATG |
| F_GAPDH | CTGGGCTACACTGAGCACC |
| R_GAPDH | AAGTGGTCGTTGAGGGCAATG |
| F_Cyclin D1 | CAATGACCCCGCACGATTTC |
| R_Cyclin D1 | CATGGAGGGCGGATTGGAA |
| F_CYLD | TCAGGCTTATGGAGCCAAGAA |
| R_CYLD | ACTTCCCTTCGGTACTTTAAGGA |

| Factor | | LUAD | | LUSC | |
|---|---|---|---|---|---|
| | | Case | Copy Number | Case | Copy Number |
| Gender | | | | | |
| | Male | 235 | -0.104±0.307 | 369 | -0.306±0.235 |
| | Female | 274 | -0.053±0.307 | 129 | -0.298±0.254 |
| Age | | | | | |
| | <60 | 135 | -0.106±0.266 | 90 | -0.341±0.223 |
| | ≥60 | 355 | -0.065±0.326 | 399 | -0.294±0.243 |
| Invasion depth | | | | | |
| | T1 | 168 | 0.279±0.022 | 292 | 0.232±0.014 |
| | T2 | 276 | 0.327±0.020 | 71 | 0.232±0.028 |
| | T3 | 44 | 0.278±0.042 | 23 | 0.268±0.056 |
| | T4 | 19 | 0.336±0.077 | 498 | 0.240±0.011 |
| Lymph node metastasis | | | | | |
| | N0 | 327 | 0.321±0.018 | 316 | 0.244±0.014 |
| | N1 | 95 | 0.312±0.032 | 131 | 0.244±0.021 |
| | N2 | 74 | 0.248±0.029 | 40 | 0.201±0.032 |
| | N3 | 2 | 0.265±0.188 | 5 | 0.234±0.105 |
| Distant metastasis | | | | | |
| | M0 | 344 | -0.081±0.291 | 408 | -0.300±0.242 |
| | M1 | 25 | -0.097±0.270 | 7 | -0.468±0.186 |
| Pathological stage | | | | | |
| | I | 273 | 0.319±0.019 | 242 | 0.250±0.016 |
| | II | 121 | 0.307±0.028 | 161 | 0.236±0.019 |
| | III | 83 | 0.271±0.030 | 84 | 0.221±0.024 |
| | IV | 25 | 0.270±0.054 | 7 | 0.186±0.070 |

**Table S3** Clinical-pathological analysis of copy number values in lung cancer

LUAD: lung adenocarcinoma; LUSC: Squamous cell carcinoma;

## 2. Supplementary Figures

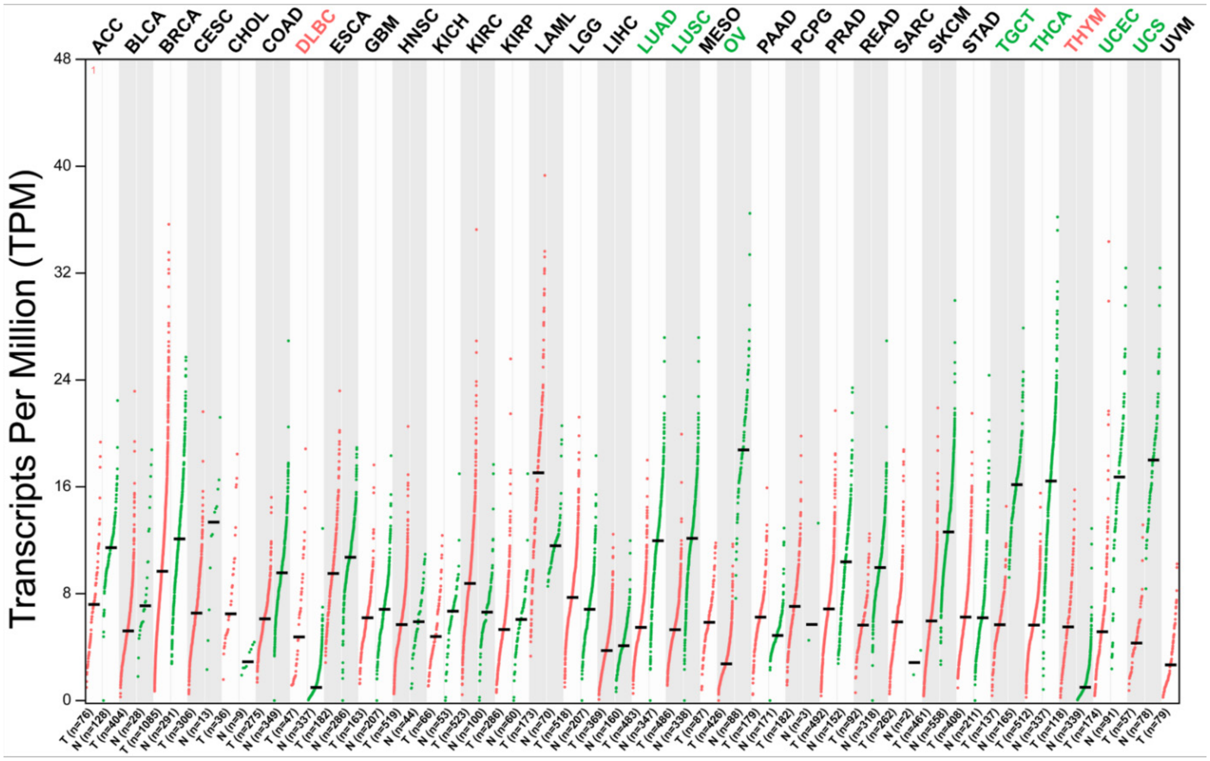


**Figure S1.** The gene expression profile across all tumor samples (T) and normal tissues (N) (dot plot). Each dot represents expression level of samples. Red dots represent tumor samples and green dots represent normal tissues. Cancer names in black indicates that there is no significant difference between tumor and normal tissues; whereas, cancer names in red or green indicate that *YTHDC2* expression either was significantly increased or decreased in tumor tissues compared with normal tissues, respectively.

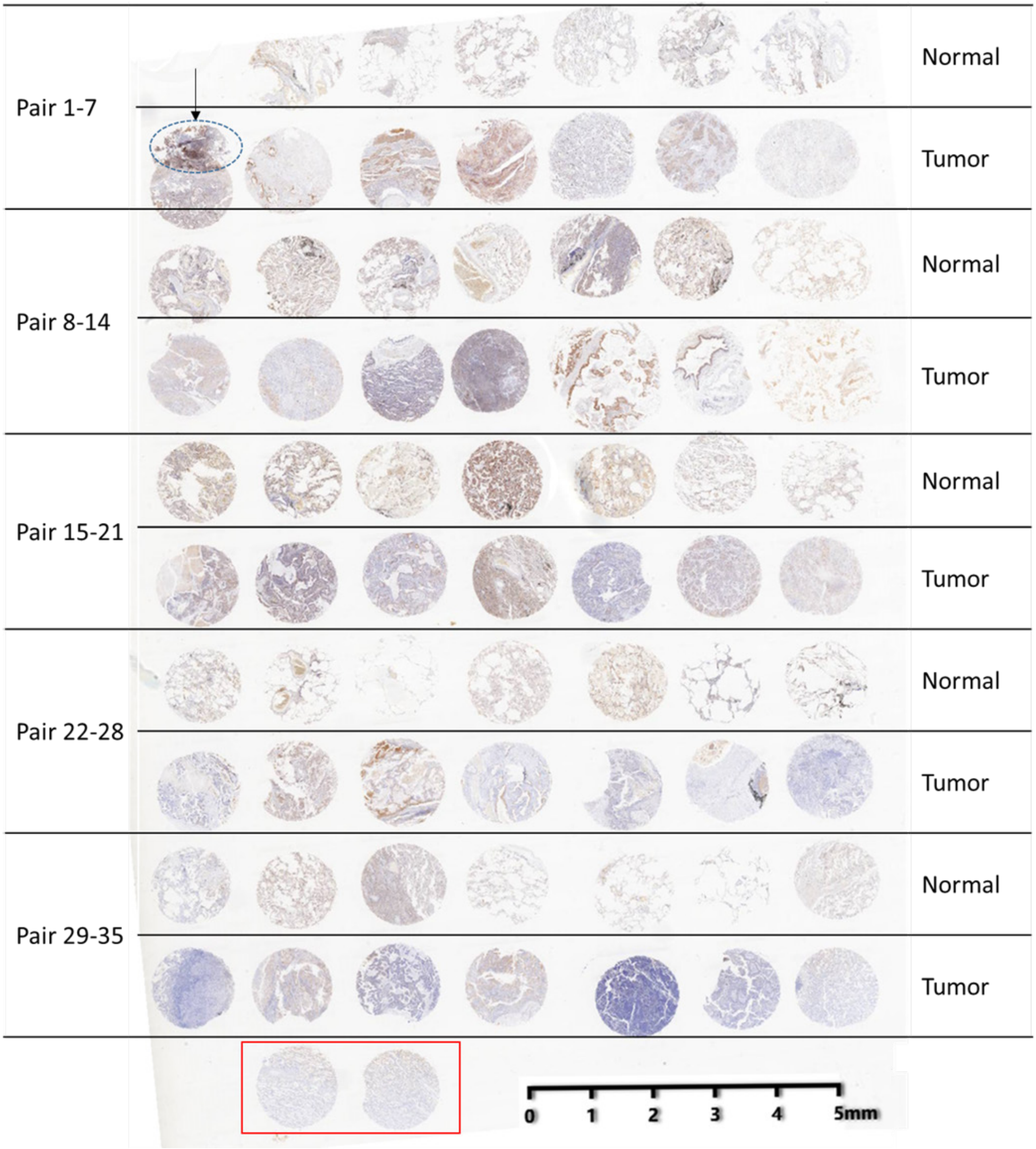


**Figure S2.** Overview of IHC staining of the lung cancer tissue array (LAC-1402). The tissues in the red box are the mark point of the chip

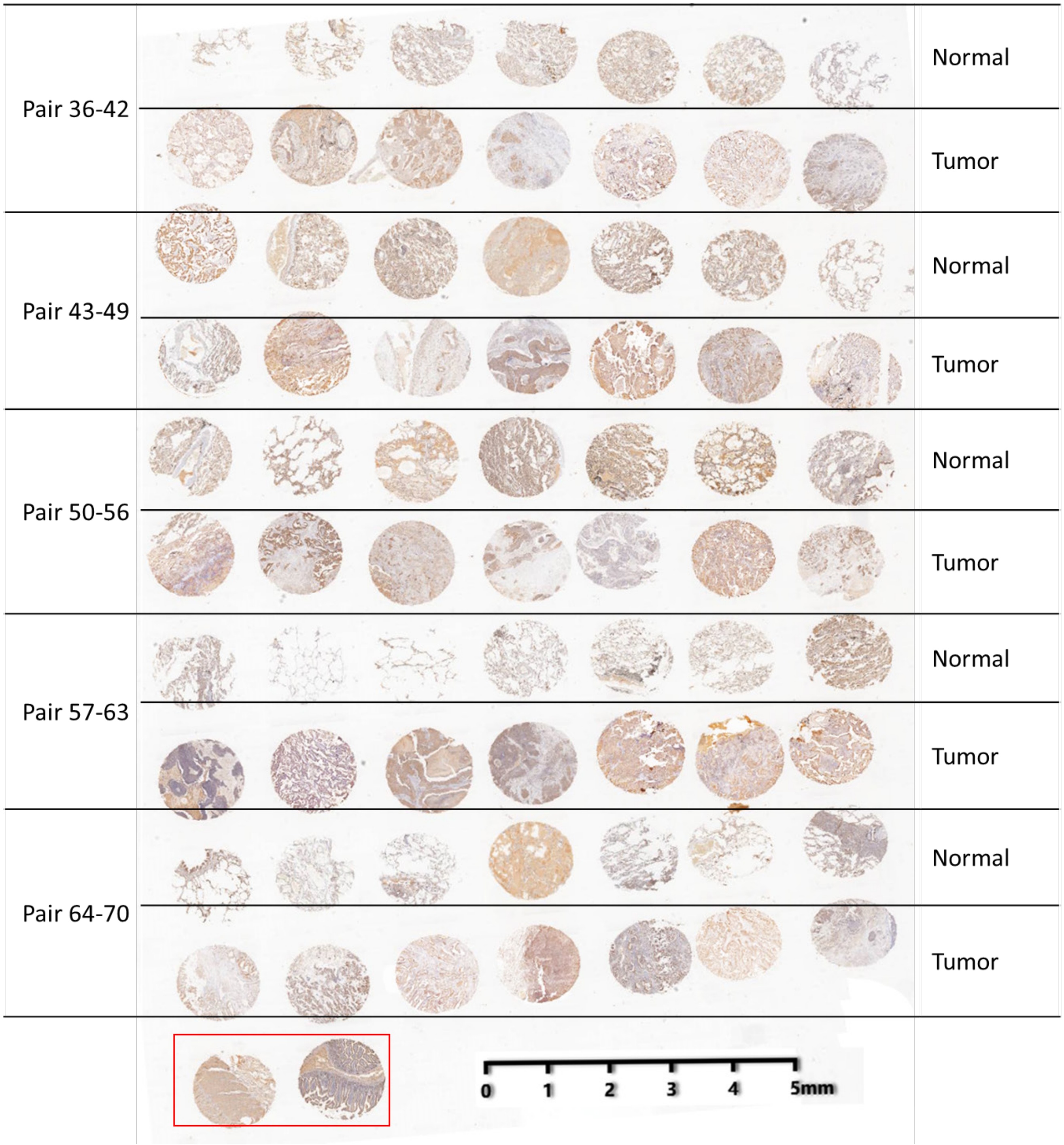


**Figure S3.** Overview of IHC staining of the lung cancer tissue array (LAC-1403). The tissues in the red box are the mark point of the chip

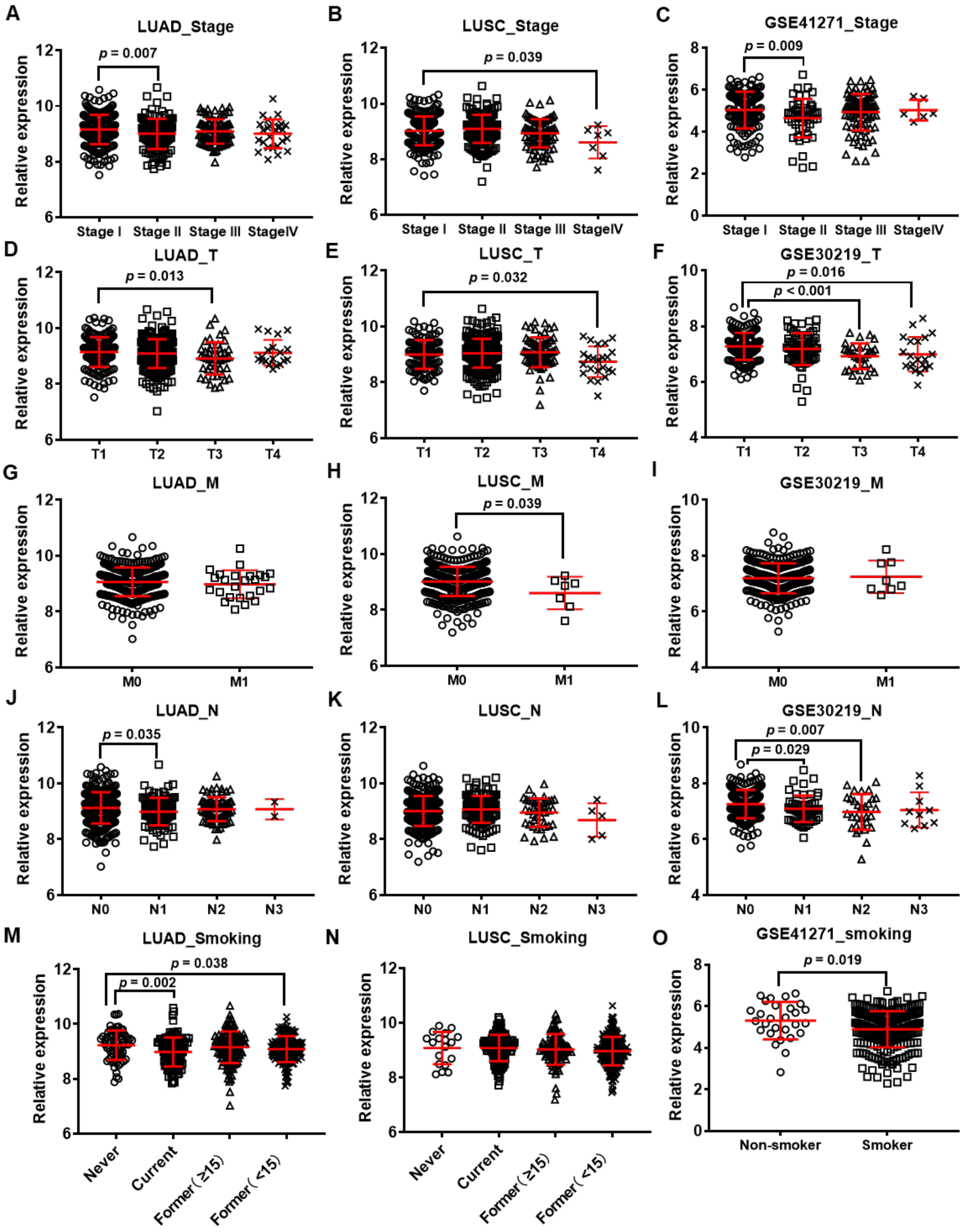


**Figure S4.** Associations between *YTHDC2* mRNA expression and pathological features. Correlation between *YTHDC2* mRNA expression and pathological stage in TCGA LUAD (A), TCGA LUSC (B), and GSE41271 (C) cohorts. Correlation between *YTHDC2* mRNA expression and pathological T stages in TCGA LUAD (D), TCGA LUSC (E), and GSE30219 (F) cohorts. Correlation between *YTHDC2* mRNA expression and pathological M stages in TCGA LUAD (G), TCGA LUSC (H), and GSE30219 (I) cohorts. Correlation between *YTHDC2* mRNA expression and pathological N stages in TCGA LUAD (J), TCGA LUSC (K), and GSE30219 (L) cohorts. Correlation between YTHDC2 mRNA expression and smoking histories in TCGA LUAD (J), TCGA LUSC (K), and GSE30219 (L) cohorts. LUAD: lung adenocarcinoma; LUSC: Squamous cell carcinoma; TCGA: The Cancer Gene Atlas.

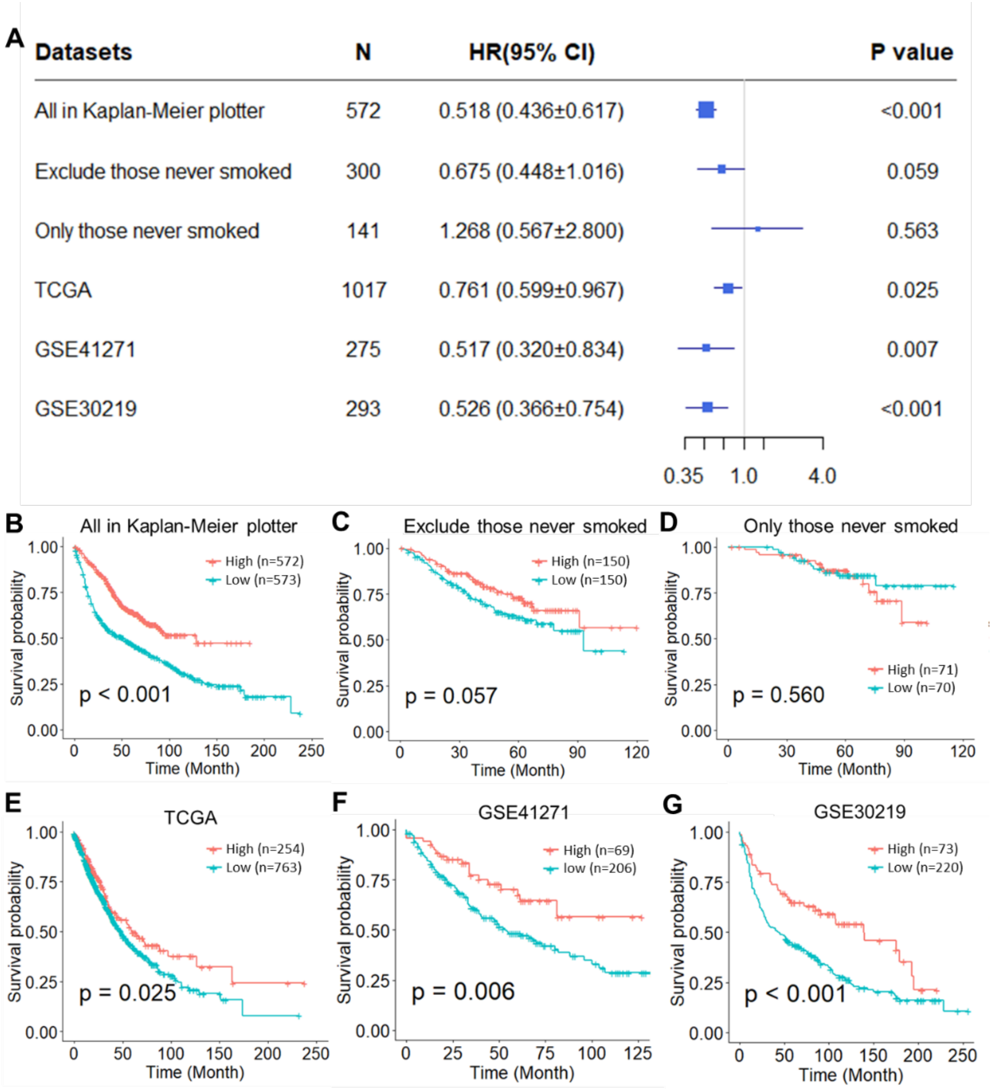


**Figure S5.** Survival analysis of *YTHDC2* in lung cancer patients. (A) Univariate Cox model analysis of the prognostic effect of *YTHDC2* expression based on lung cancer datasets. Kaplan-Meier analysis and survival curve were performed based on the Kaplan-Meier plotter datasets of (B) all lung cancer patients, (C) exclude those lung cancer patients who never smoked, and (D) only those lung cancer patients who never smoked, in addition to datasets from (E) TCGA, (F) GSE41271, and (G) GSE30219. The endpoint was overall survival (OS). The mRNA expression level of *YTHDC2* was divided into two groups based on their upper quartiles. TCGA: The Cancer Gene Atlas.

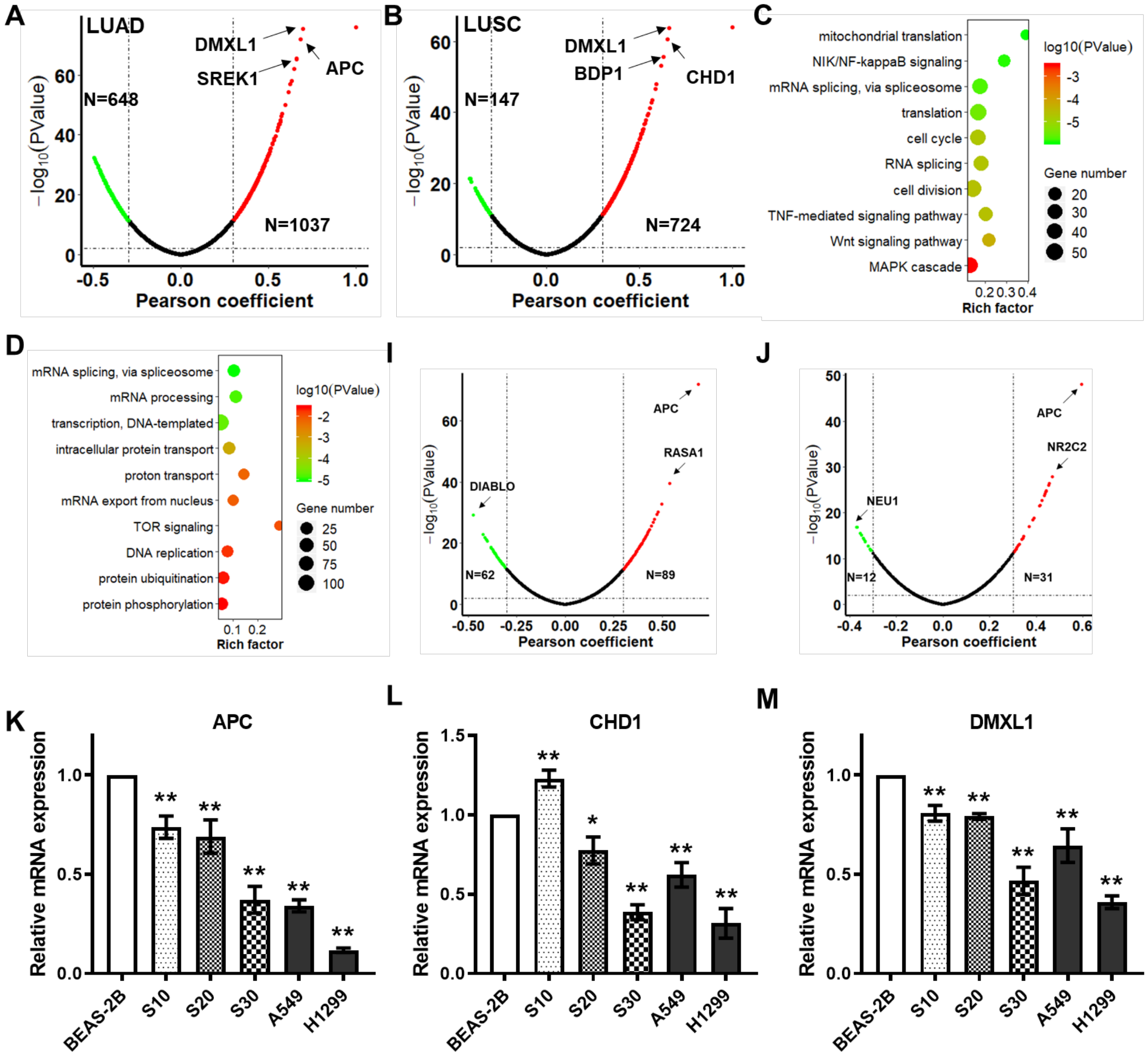


**Figure S6.** Identification of TSGs and MRGs in *YTHDC2* related genes. Volcano plots showing the genes correlated with *YTHDC2* in LUAD (A) and LUSC (B) in the TCGA database by using the LinkedOmics online tool. Bubble chart showing the biological process enrichment results of the genes associated with LUAD (C) and LUSC (D). Rich Factor is the ratio of the related genes annotated in a pathway to all the genes in this pathway. The larger the Rich factor, the greater the degree of enrichment. The color gradient from red to green represents the *p*-value; the closer to green color, the lower the *p*-value and the higher the significance level corresponding to the enrichment. Volcano plots showing the MRGs in *YTHDC2* related genes in LUAD (E) and LUSC (F). The red dots in volcano plots represent the positively related genes (Pearson coefficient > 0.3, $p < 0.001$), and the green dots represent the negatively related genes (Pearson coefficient < -0.3, $p < 0.001$), while the black dots represent genes have no significant correlation with *YTHDC2* (Pearson coefficient between -0.3 and 0.3). The relative mRNA expression level of APC (G), CHD1 (H), DMXL1 (I) in cigarette smoke-exposed cells (S10, S20 and S30) and normal BEAS-2B cells. TSGs: Tumor suppressor genes; MRGs: Metastasis related genes; TCGA: The Cancer Gene Atlas; LUAD: Lung adenocarcinoma; LUSC: Lung squamous cell carcinoma.

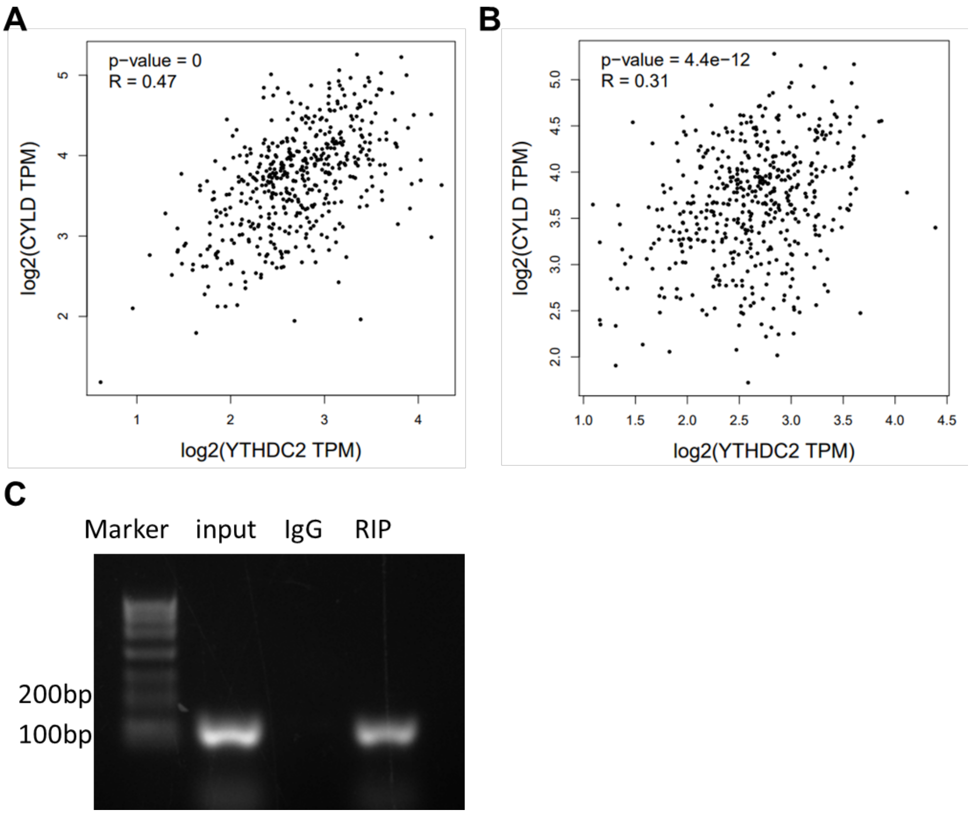


**Figure S7.** Correlation and RIP PCR analysis. Pearson correlation analysis showed a significant positive correlation between YTHDC2 and CYLD mRNA expression in LUAD (A) and LUSC (B). (C) RIP PCR assay showed a significant enrichment of CYLD mRNA in YTHDC2 RIP sample. Anti-IgG antibody is used as control.

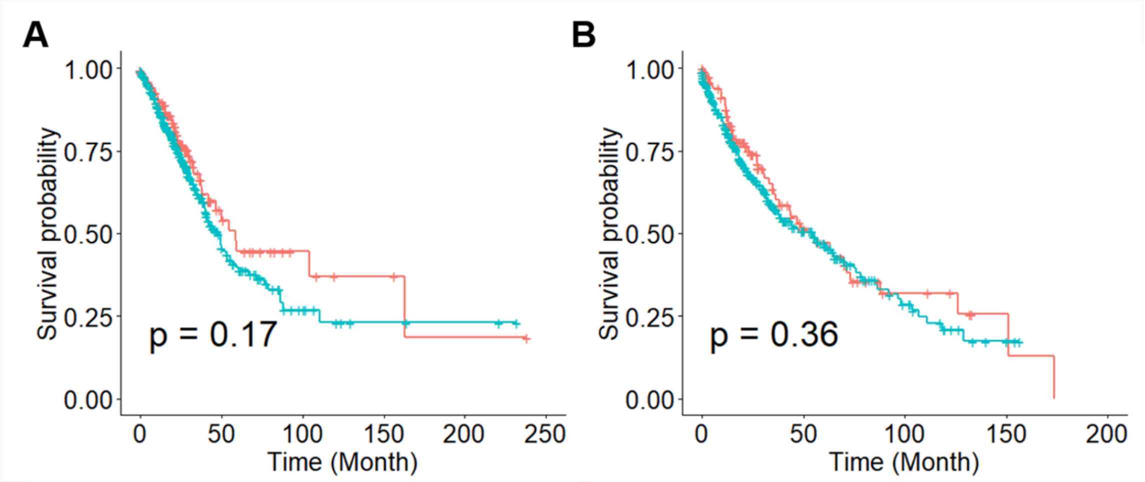


**Figure S8.** Kaplan-Meier analysis of *YTHDC2* copy numbers in lung cancer patients in TCGA_LUAD (A) and TCGA_LUSC (B) datasets. LUAD: lung adenocarcinoma; LUSC: Squamous cell carcinoma; TCGA: The Cancer Gene Atlas.

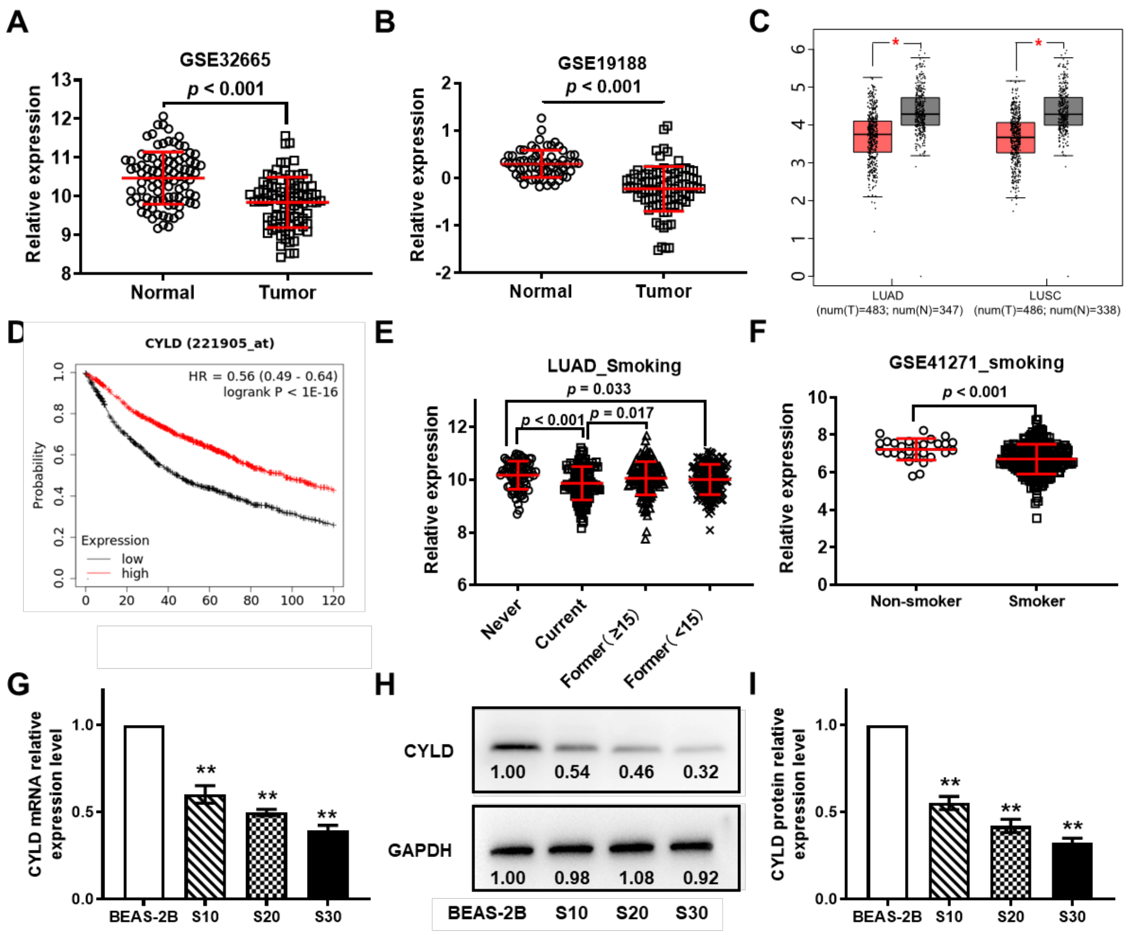


**Figure S9.** Differential analysis of *CYLD* mRNA expression in lung cancer tissues from (A) GSE32665 and (B) GSE19188 datasets. (C) Differential analysis of *CYLD* mRNA expression in lung cancer tissues based on the Gene Expression Profiling Interactive Analysis tool. *P<0.05 vs. normal tissues. (D) Kaplan-Meier curve were plotted using Kaplan-Meier Plotter online tool. (E) *CYLD* mRNA expression in lung cancer tissues with different smoking histories in TCGA_LUAD dataset. (F) *CYLD* mRNA expression in lung cancer tissues with different smoking histories in GSE41271 dataset. (G) Relative mRNA expression level of *CYLD* in CS-exposed cells (S10, S20 and S30) and normal BEAS-2B cells. Western blot analysis (H) and quantitative results (I) of CYLD protein expression in CS-exposed cells (S10, S20 and S30) and normal BEAS-2B cells. S10, S20 and S30 represent BEAS-2B cells exposed to CS for 10, 20 and 30 passages, respectively. **P<0.01 vs. normal BEAS-2B cells. CS, cigarette smoke; TCGA: The Cancer Genome Atlas; LUAD**,** lung adenocarcinoma.
